# Supplementary material for: A tunable dual-input system for on-demand dynamic gene expression regulation
Source: Nat Commun. 2019 Oct 2;10:4481. doi: 10.1038/s41467-019-12329-9 (PMC6775159; doi:10.1038/s41467-019-12329-9)
Supplement: Supplementary file 9 — Supplementary Information [file 41467_2019_12329_MOESM9_ESM.pdf]

# A tunable dual input system for ‘on-demand’ dynamic gene expression regulation

Elisa Pedone, Lorena Postiglione, Francesco Aulicino, Dan L. Rocca, Sandra Montes-Olivas, Mahmoud Khazim, Diego di Bernardo, Maria Pia Cosma, Lucia Marucci .

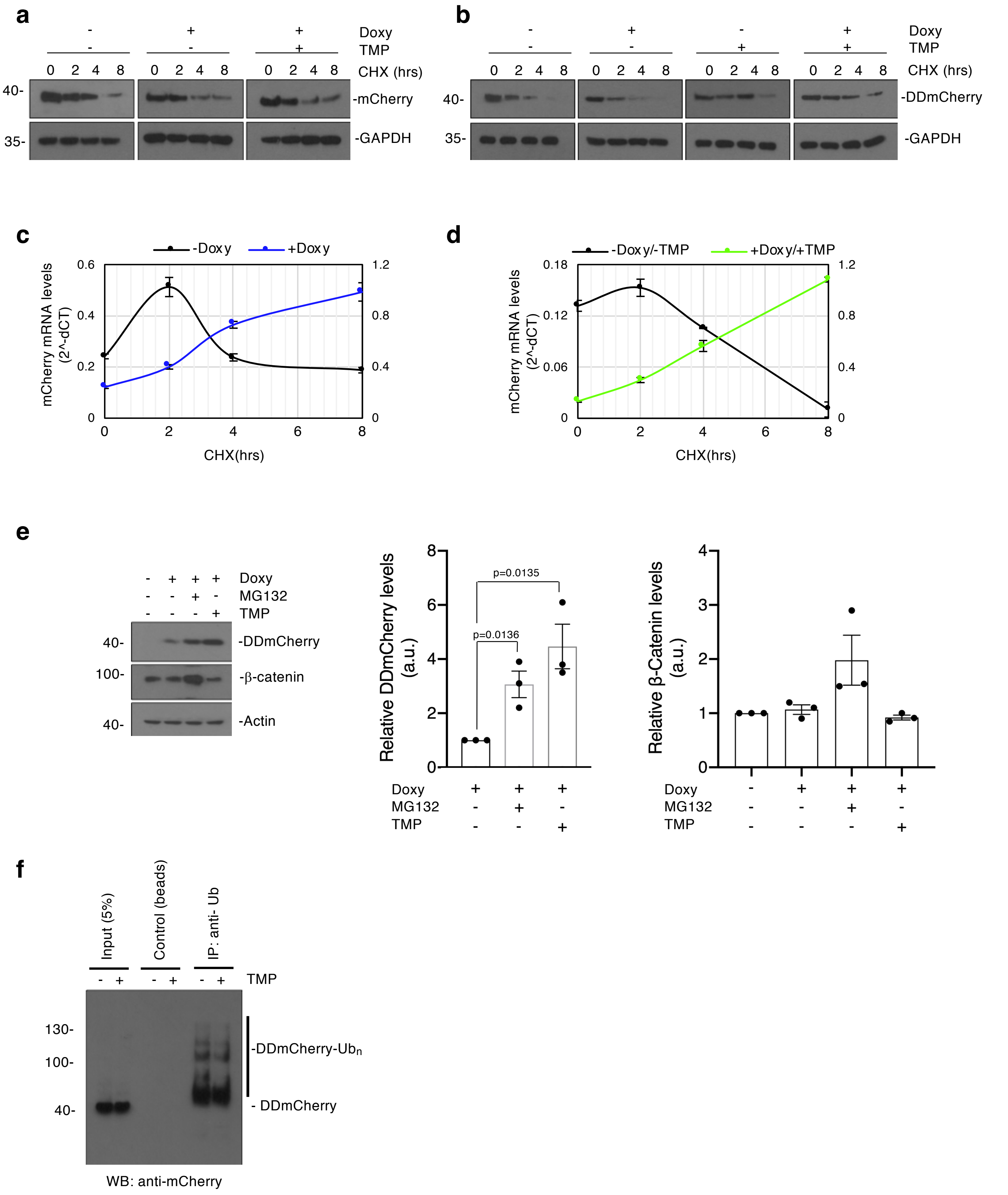


**Supplementary Figure 1**

**Supplementary Figure 1: TMP mechanism of action**

(a-d) mCherry and DDmCherry protein (a, b) and mRNA (c, d) levels measured by western-blot and qPCR, respectively, in EF1a-rtTA_TRE3G-mCherry (a, c) and EF1a-rtTA_TRE3G-DDmCherry (b, d) mESCs, treated as indicated in Fig. 1e and relative legend. (e) EF1a-rtTA_TRE3G-DDmCherry mESCs were incubated with Doxy (1000ng/mL) and either TMP (10μM) or MG132 (5μM) for 16hrs. Blocking proteosomal degradation enabled to effectively mimic the stabilising effect of TMP specifically on DDmCherry but not on endogenous β-catenin used as control. Western-blot densitometric quantifications are shown (e, inset). Plotted DDmCherry and β-catenin values are normalised against the housekeeping gene Actin. (f) Ubiquitination status analysis. EF1a-rtTA_TRE3G-DDmCherry mESCs were incubated for 24hrs with Doxy (1000ng/mL) and TMP (100nM), washed and incubated with or without TMP (100nM) for additional 12hrs. Samples were processed as indicated in the Methods, immunoprecipitated with an anti-ubiquitin antibody and blotted with the mCherry antibody to analyse the ubiquitination status of the DDmCherry protein. Note the decrease in poly-ubiquitinated species of DDmCherry when TMP is present. Data are means ± SEM (n=3, e inset). p values from two-tailed unpaired t test are shown. Source data are provided as a Source Data file.

**a**

| EF1a-rtTA TRE3G-mCherry | Doxy Titration | |
|---|---|---|
| Doxy | Median Fluorescence Intensity (MFI) | % mCherry+ Cells |
| - | 515±3.2 | 0.08±0.031 |
| 1ng/mL | 539±13 | 0.175±0.035 |
| 5ng/mL | 598.67±15.2 | 3.05±0.63 |
| 50ng/mL | 5719.7±30.7 | 75.77±0.5 |
| 100ng/mL | 7388.7±294.7 | 81.77±0.67 |
| 500ng/mL | 8961.7±102.2 | 89.23±0.088 |
| 1000ng/mL | 8987.7±76.7 | 89.83±0.48 |

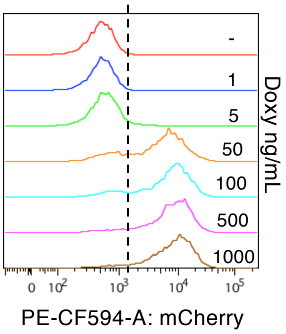


**b**

| EF1a-rtTA TRE3G-DDmCherry | | Doxy/TMP Titration | |
|---|---|---|---|
| Doxy | TMP | Median Fluorescence Intensity (MFI) | % mCherry+ Cells |
| - | 10μM | 286.33±4.7 | 0.417±0.1 |
| 1ng/mL | 10μM | 310.67±20.2 | 0.23±0.08 |
| 5ng/mL | 10μM | 292±2.6 | 0.35±0.036 |
| 50ng/mL | 10μM | 3762.33±33.2 | 80.1±0.47 |
| 100ng/mL | 10μM | 7478.33±148.2 | 93±0.38 |
| 500ng/mL | 10μM | 9136.33±283.3 | 96.5±0.34 |
| 1000ng/mL | 10μM | 9041±224.5 | 97.17±0.26 |
| 1000ng/mL | - | 1812±33 | 69.4±0.7 |
| 1000ng/mL | 1nM | 2829.33±83.5 | 81.47±0.7 |
| 1000ng/mL | 10nM | 6662.33±58.7 | 95.1±0.26 |
| 1000ng/mL | 100nM | 8546.7±203.7 | 97.1±0.15 |
| 1000ng/mL | 1μM | 8541±36.1 | 96.57±0.17 |
| 1000ng/mL | 10μM | 8650.33±297.4 | 96.53±0.03 |

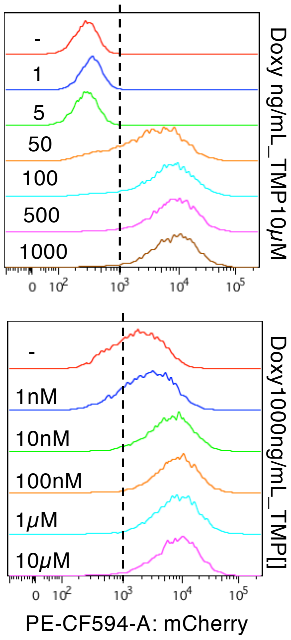


**c**

| EF1a-rtTA TRE3G-mCherry | Dynamic Time-Course | |
|---|---|---|
| Doxy | Median Fluorescence Intensity (MFI) | % mCherry+ Cells |
| - | 482.5±2.5 | 0.28±0.09 |
| 14hrs 1000ng/mL | 6383±328.4 | 84.2±1.17 |
| 5hrs wash-out | 3466±204.7 | 70.3±1.09 |
| 8hrs wash-out | 3566.5±333 | 69.47±2.2 |
| 24hrs wash-out | 562±26.6 | 3.36±0.85 |

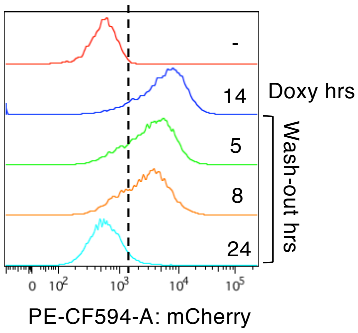


**Supplementary Figure 2**

**d**

| EF1a-rtTA TRE3G-DDmCherry | | Dynamic Time-Course | |
|---|---|---|---|
| **Doxy** | **TMP** | **Median Fluorescence Intensity (MFI)** | **% mCherry+ Cells** |
| - | - | 413.5±14.5 | 0.365±0.055 |
| 14hrs 1000ng/mL | - | 1821.7±101 | 45.8±2.9 |
| 5hrs wash-out | | 785.33±36.8 | 11.22±0.8 |
| 8hrs wash-out | | 737±65.6 | 10.27±2.6 |
| 24hrs wash-out | | 316±3.6 | 0.61±0.07 |
| - | 100nM | 408.5±9.5 | 0.305±0.095 |
| 14hrs 1000ng/mL | 100nM | 6669±214.5 | 89.6±0.72 |
| 5hrs wash-out | | 1839.33±62 | 46.6±1.73 |
| 8hrs wash-out | | 1427±38.9 | 33.33±1.5 |
| 24hrs wash-out | | 364.67±17.2 | 1.21±0.047 |

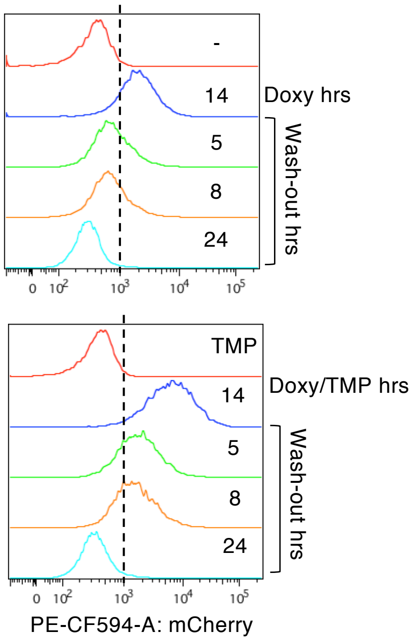


**e**

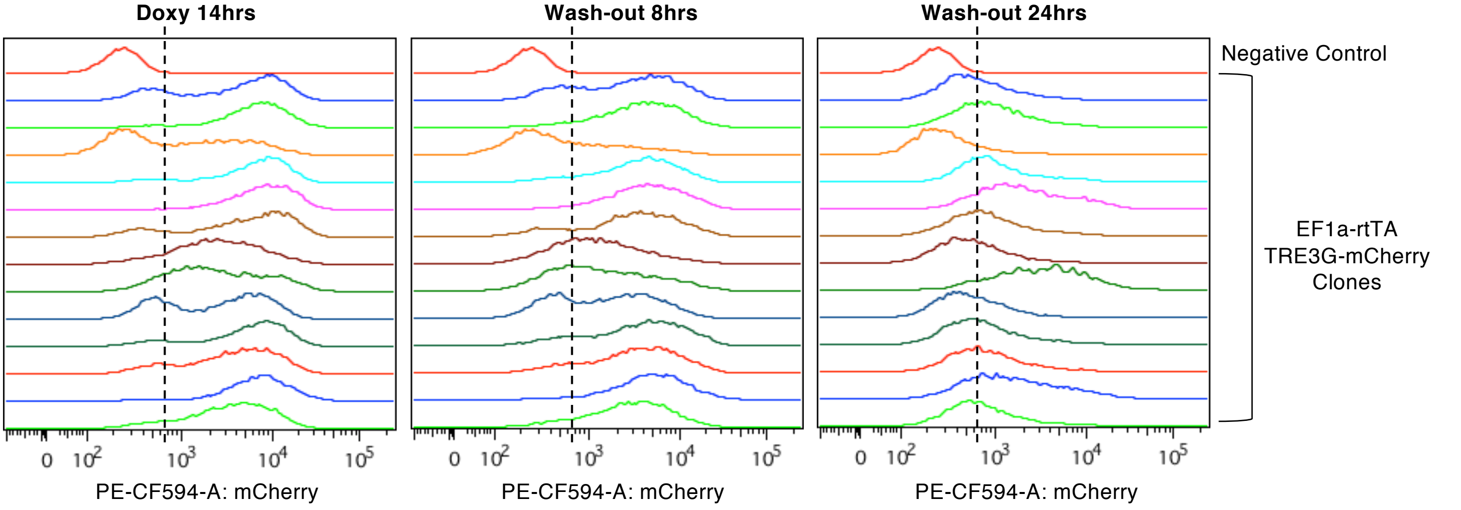


**f**

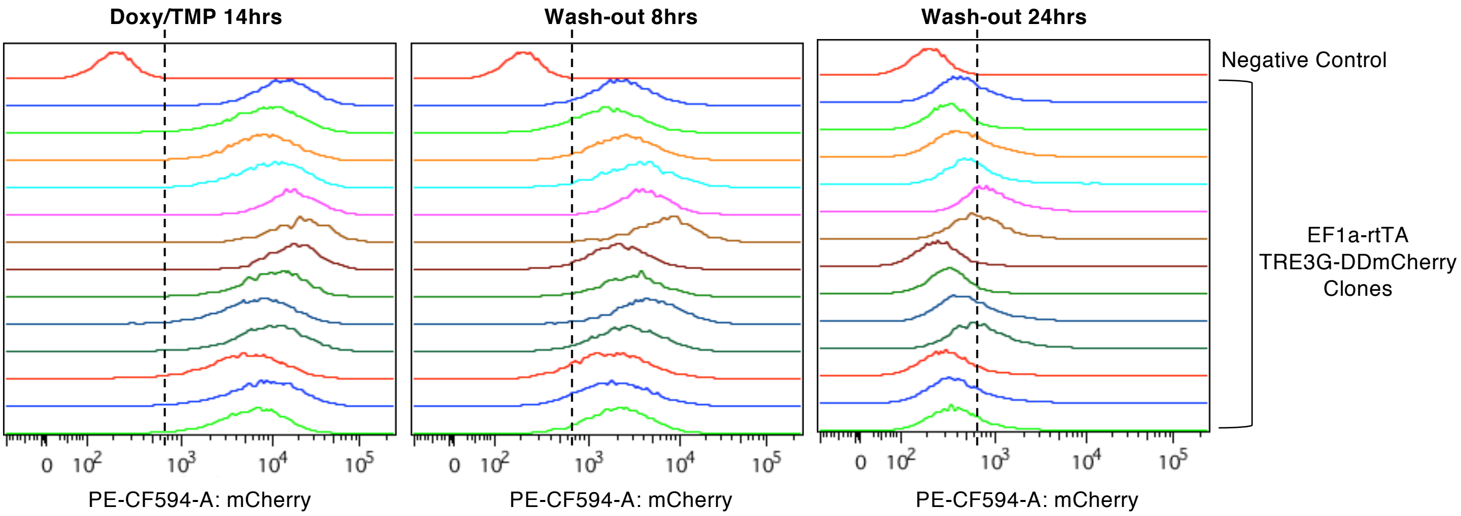


**Supplementary Figure 2**

**g**

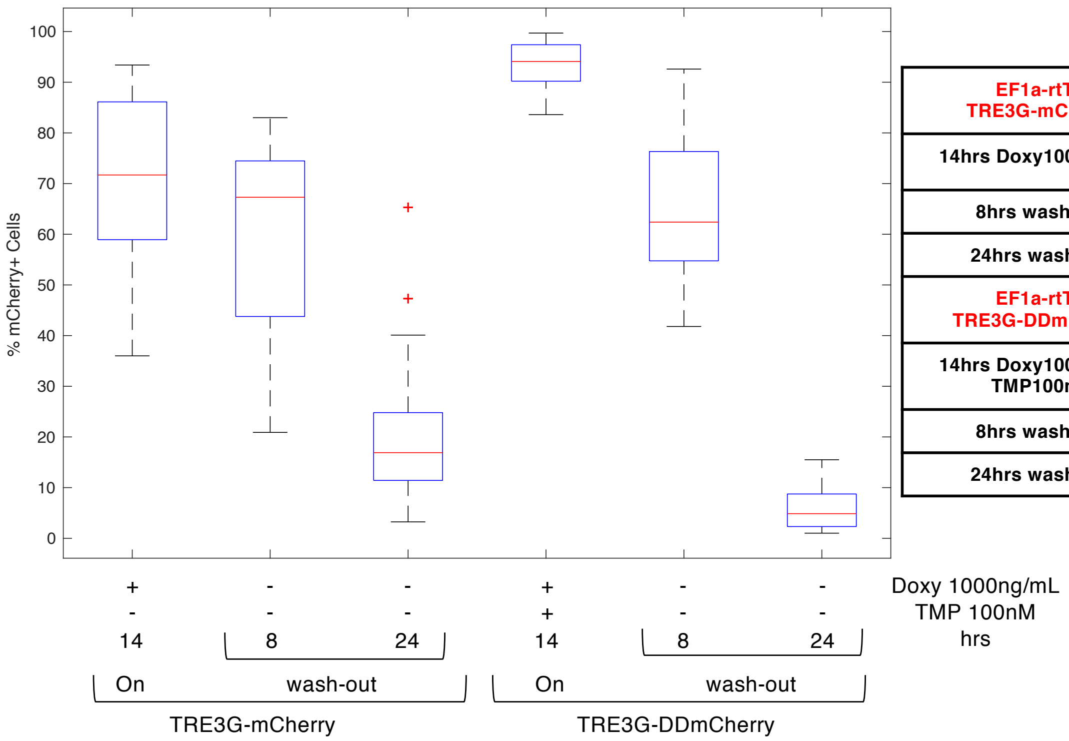


| EF1a-rtTA TRE3G-mCherry | Coefficient of Variation (CV) of the mCherry+ Cells |
|---|---|
| 14hrs Doxy1000ng/mL | 0.2307 |
| 8hrs wash-out | 0.3217 |
| 24hrs wash-out | 0.8076 |
| **EF1a-rtTA TRE3G-DDmCherry** | **Coefficient of Variation (CV) of the mCherry+ Cells** |
| 14hrs Doxy1000ng/mL TMP100nM | 0.0498 |
| 8hrs wash-out | 0.2417 |
| 24hrs wash-out | 0.7334 |

**h**

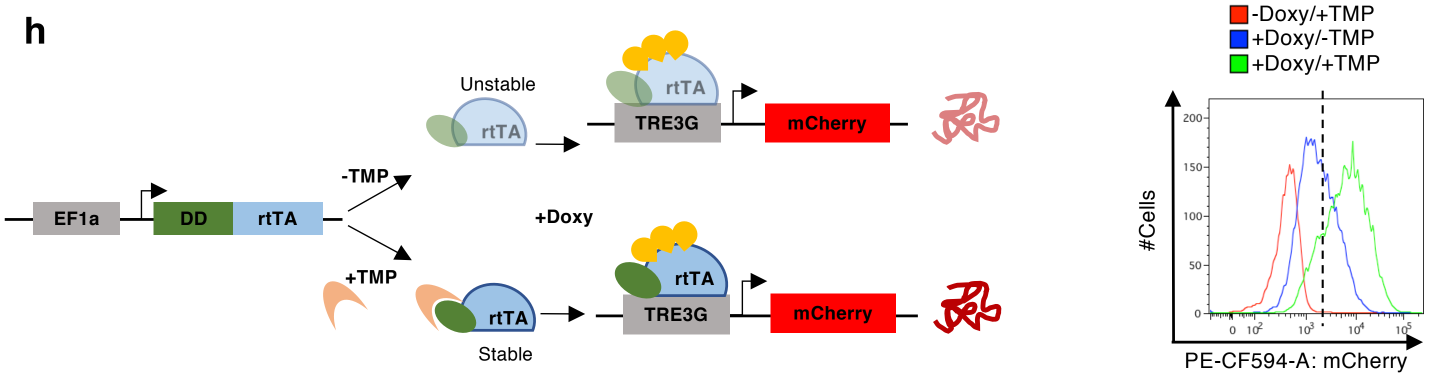


**i**

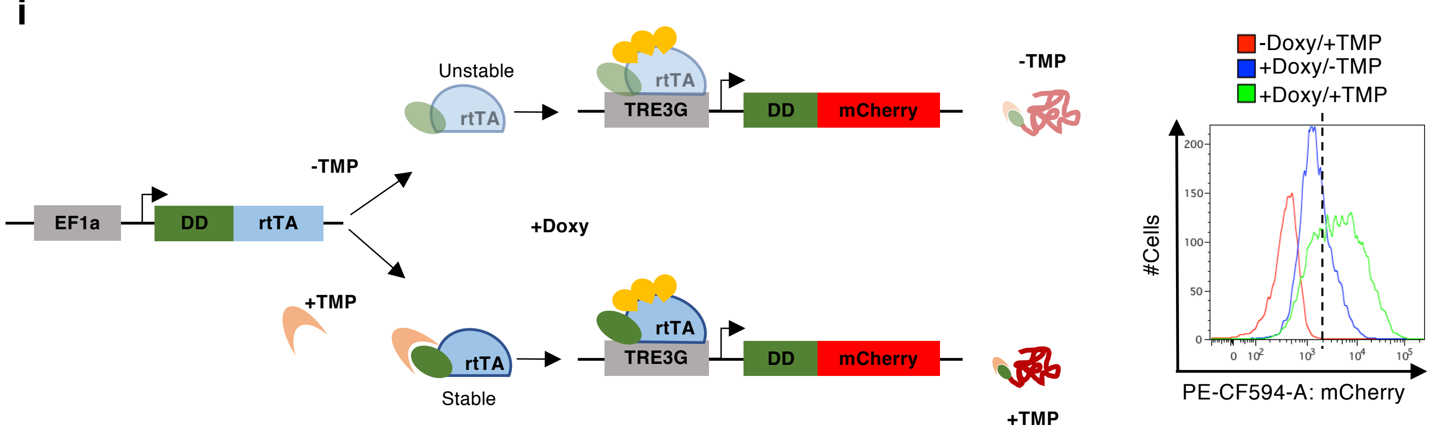


**Supplementary Figure 2**

**j**

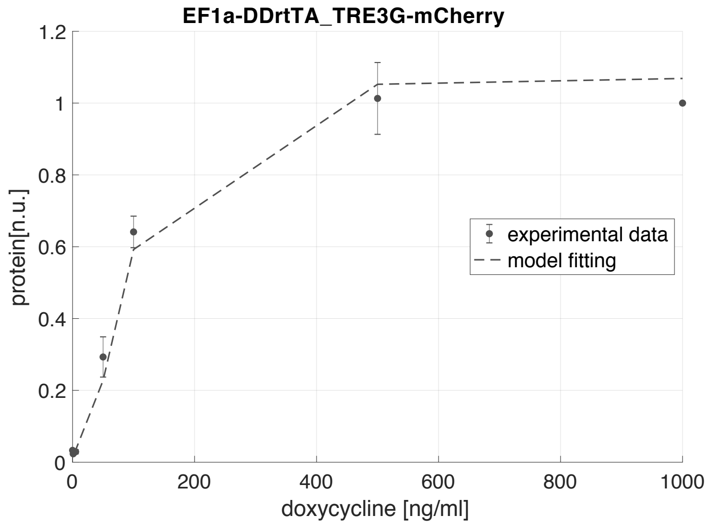


**k**

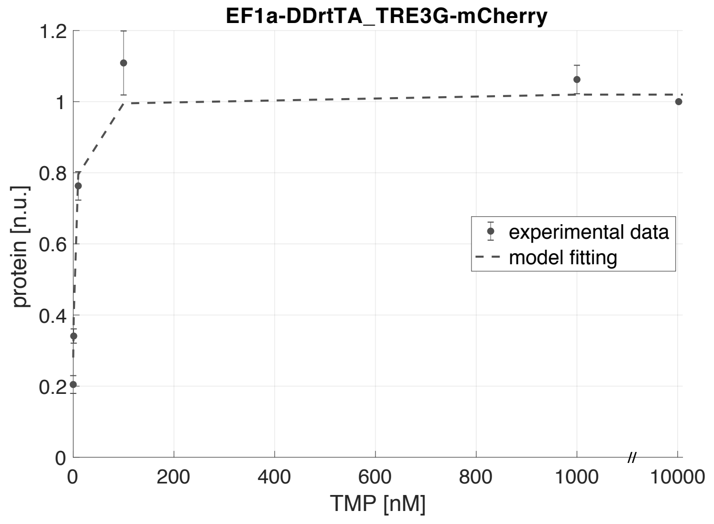


**l**

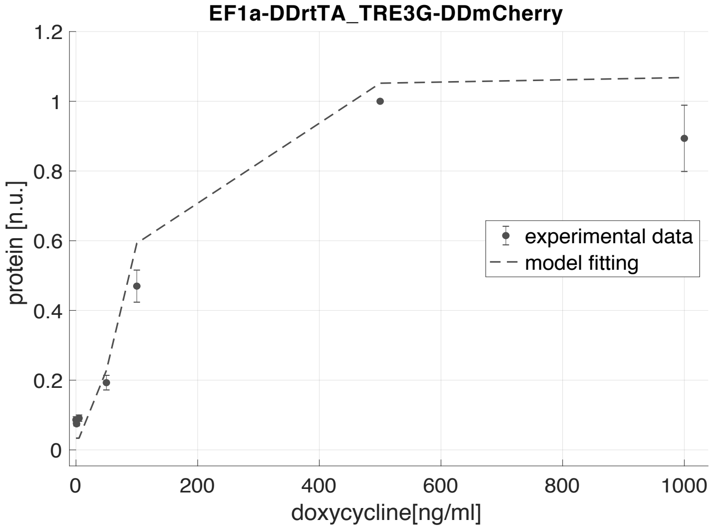


**m**

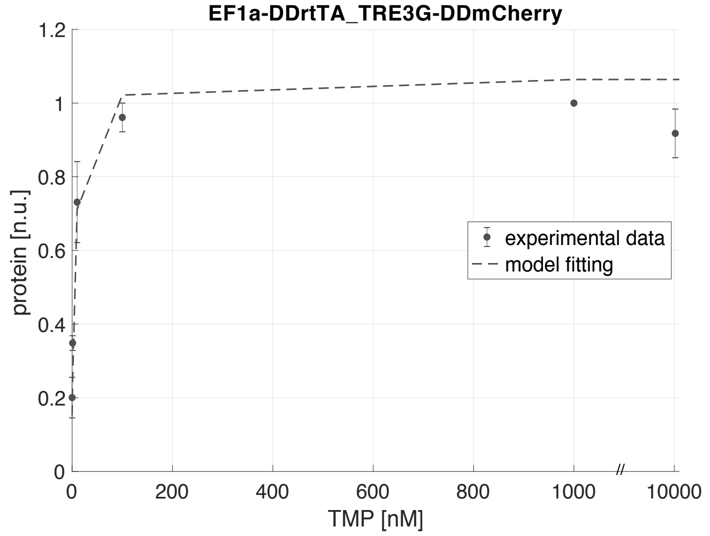


**n**

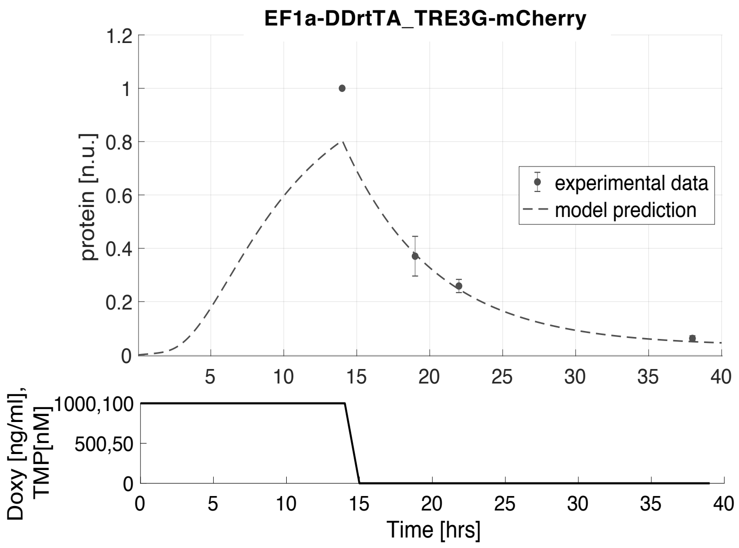


**o**

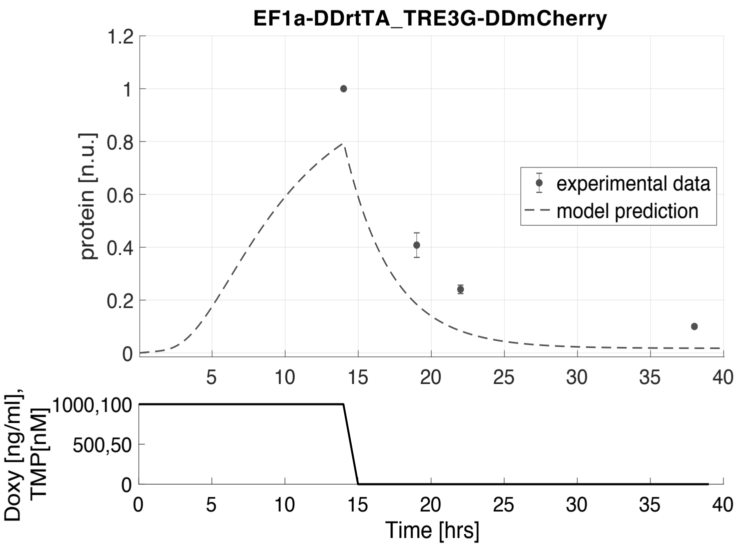


**Supplementary Figure 2**

**p**

| EF1a-DDrtTA TRE3G-mCherry | | Doxy/TMP Titration | |
|---|---|---|---|
| Doxy | TMP | Median Fluorescence Intensity (MFI) | % mCherry+ Cells |
| - | 10$\mu$M | 546±12.3 | 0.433±0.085 |
| 1ng/mL | 10$\mu$M | 386.7±6.2 | 0.082±0.02 |
| 5ng/mL | 10$\mu$M | 481±36.1 | 0.42±0.071 |
| 50ng/mL | 10$\mu$M | 4848.33±352 | 66.37±1.3 |
| 100ng/mL | 10$\mu$M | 10610.7±292.6 | 80±0.6 |
| 500ng/mL | 10$\mu$M | 16760.7±353 | 90.3±0.63 |
| 1000ng/mL | 10$\mu$M | 16546.33±600 | 91.3±0.78 |
| 1000ng/mL | - | 2955±193.5 | 63±2.2 |
| 1000ng/mL | 1nM | 4924±100 | 74.2±0.8 |
| 1000ng/mL | 10nM | 11024.7±210 | 87±0.2 |
| 1000ng/mL | 100nM | 16017.7±754.3 | 91.6±0.68 |
| 1000ng/mL | 1$\mu$M | 15344.7±241 | 91.3±0.18 |
| 1000ng/mL | 10$\mu$M | 14445±146.5 | 90.6±0.09 |

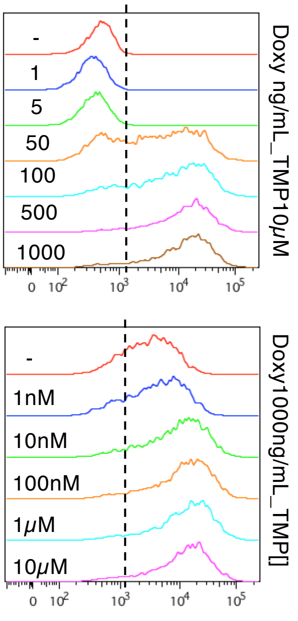


**q**

| EF1a-DDrtTA TRE3G-DDmCherry | | Doxy/TMP Titration | |
|---|---|---|---|
| Doxy | TMP | Median Fluorescence Intensity (MFI) | % mCherry+ Cells |
| - | 10$\mu$M | 553.33±10.7 | 0.159±0.05 |
| 1ng/mL | 10$\mu$M | 479.33±43.4 | 0.128±0.032 |
| 5ng/mL | 10$\mu$M | 583.67±19.8 | 0.23±0.09 |
| 50ng/mL | 10$\mu$M | 1237±88.6 | 38.7±1.9 |
| 100ng/mL | 10$\mu$M | 3013±177.1 | 59±1.3 |
| 500ng/mL | 10$\mu$M | 6413±347 | 77.3±0.51 |
| 1000ng/mL | 10$\mu$M | 5731±116 | 75.1±0.32 |
| 1000ng/mL | - | 1221.67±132 | 33.8±2.5 |
| 1000ng/mL | 1nM | 2123.33±137.6 | 52.2±2.1 |
| 1000ng/mL | 10nM | 4455±200 | 71.3±0.43 |
| 1000ng/mL | 100nM | 5856±146.5 | 76.13±0.75 |
| 1000ng/mL | 1$\mu$M | 6093±327.6 | 79.57±2.67 |
| 1000ng/mL | 10$\mu$M | 5592±58 | 75.8±0.26 |

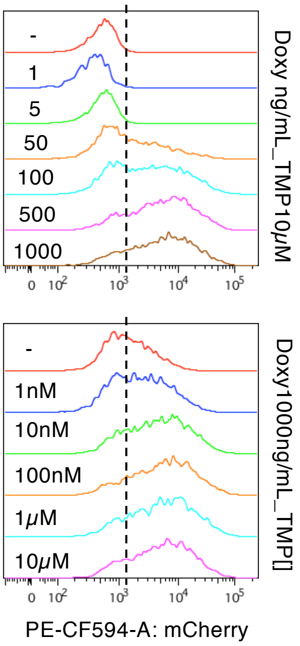

**r**

| EF1a-DDrtTA TRE3G-mCherry | | Dynamic Time-Course | |
|---|---|---|---|
| **Doxy** | **TMP** | **Median Fluorescence Intensity (MFI)** | **% mCherry+ Cells** |
| - | - | 444±14 | 0.255±0.095 |
| 14hrs 1000ng/mL | - | 1660±50.7 | 43.1±1 |
| 5hrs wash-out | | 847.33±38.8 | 19.33±2 |
| 8hrs wash-out | | 758±45.1 | 15.8±2 |
| 24hrs wash-out | | 332.67±3.5 | 1.45±0.18 |
| - | 100nM | 444.5±20.5 | 0.715±0.015 |
| 14hrs 1000ng/mL | 100nM | 6386±323 | 83.1±1.3 |
| 5hrs wash-out | | 2515±138.8 | 58.77±1.9 |
| 8hrs wash-out | | 1756±139.5 | 45.5±2.9 |
| 24hrs wash-out | | 421.33±17.3 | 2.65±0.16 |

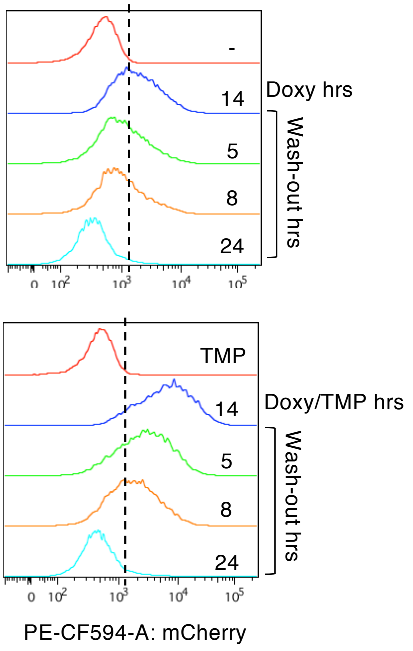


**s**

| EF1a-DDrtTA TRE3G-DDmCherry | | Dynamic Time-Course | |
|---|---|---|---|
| **Doxy** | **TMP** | **Median Fluorescence Intensity (MFI)** | **% mCherry+ Cells** |
| - | - | 428.5±9.5 | 0.94±0.06 |
| 14hrs 1000ng/mL | - | 1256±127.5 | 29.5±2.6 |
| 5hrs wash-out | | 728.33±16.7 | 9.15±0.13 |
| 8hrs wash-out | | 586.33±3 | 4.72±0.39 |
| 24hrs wash-out | | 340.67±3.3 | 0.99±0.1 |
| - | 100nM | 421±1 | 0.43±0.16 |
| 14hrs 1000ng/mL | 100nM | 4149±45.6 | 71.3±1.3 |
| 5hrs wash-out | | 1695±96.5 | 44.3±2.4 |
| 8hrs wash-out | | 999.67±29.6 | 23.7±1.16 |
| 24hrs wash-out | | 416±14.3 | 2.89±0.13 |

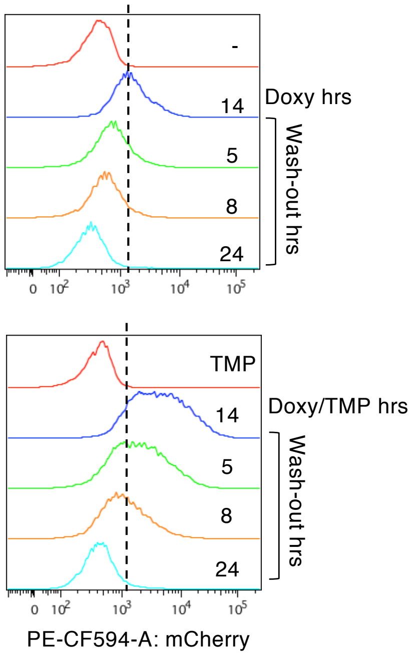

t

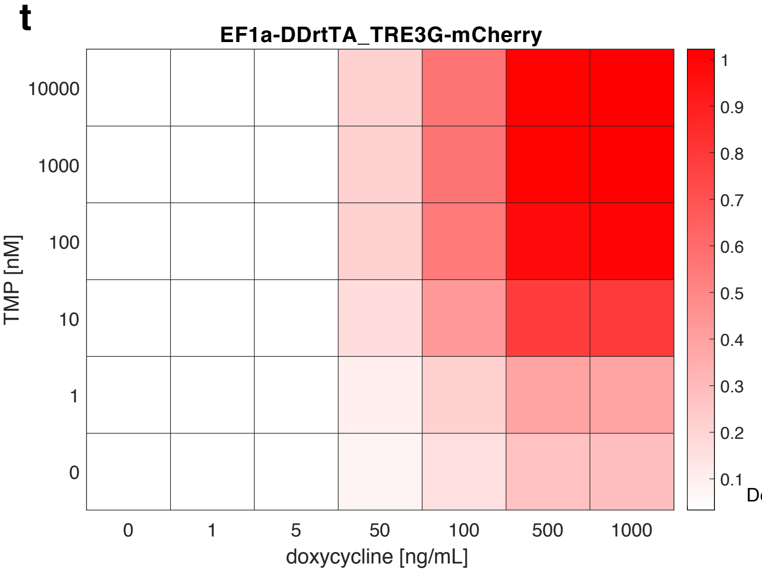


u

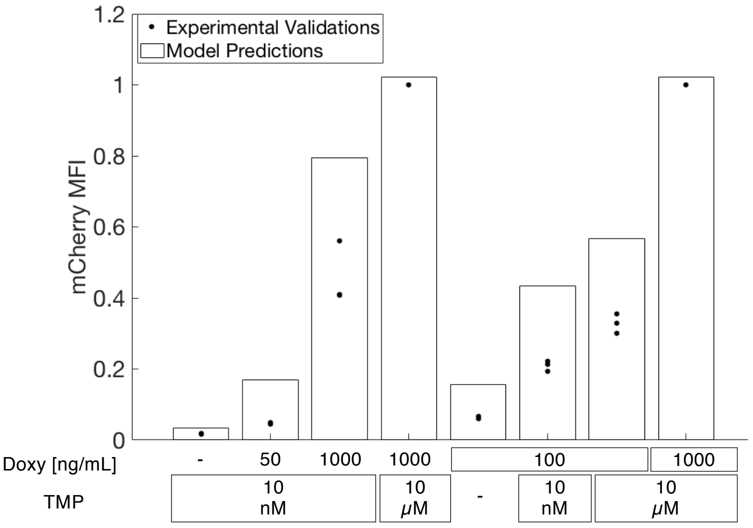


v

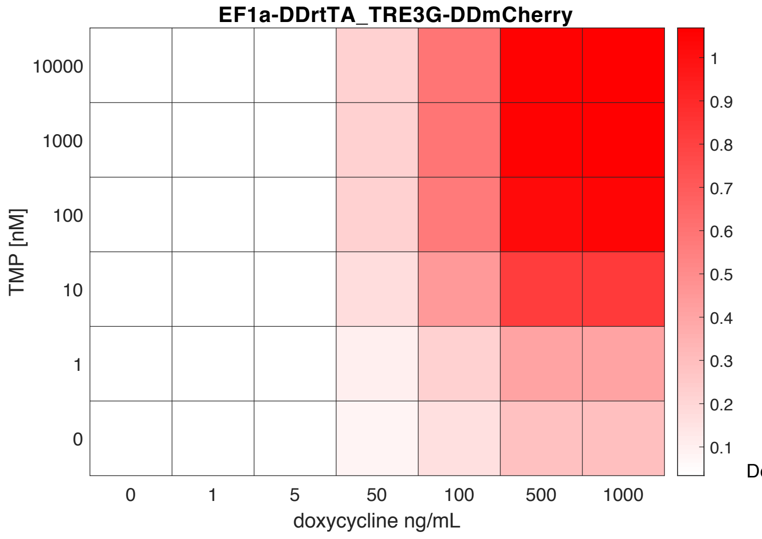


w

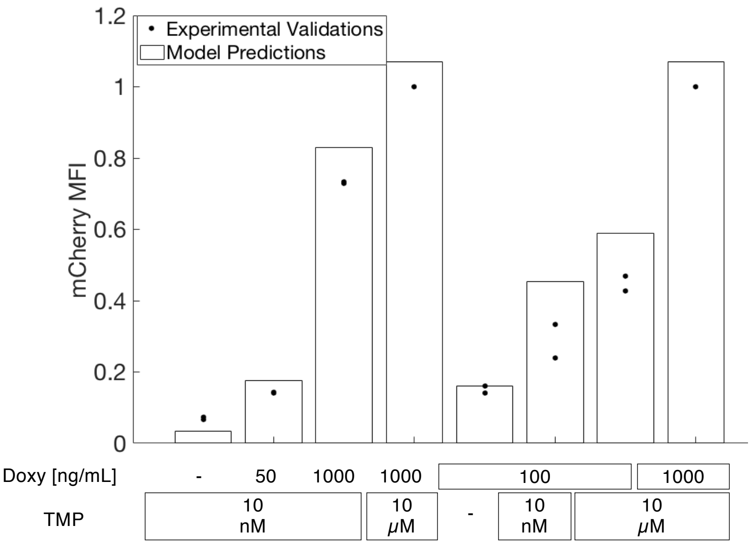


x

| HeLa_EF1a-rtTA TRE3G-DDmCherry | | Doxy/TMP Titration | |
|---|---|---|---|
| Doxy | TMP | Median Fluorescence Intensity (MFI) | % mCherry+ Cells |
| - | 10μM | 520±8.4 | 1±0.1 |
| 1ng/mL | 10μM | 550±3.5 | 1±0.3 |
| 5ng/mL | 10μM | 1625±35.6 | 33.3±1 |
| 50ng/mL | 10μM | 13858±308.6 | 78±0.3 |
| 100ng/mL | 10μM | 16676±108.4 | 80.9±0.5 |
| 500ng/mL | 10μM | 17958±84.2 | 81.9±0.8 |
| 1000ng/mL | 10μM | 18145±127.6 | 82.8±0.5 |
| 1000ng/mL | - | 3786±115.8 | 57.5±1 |
| 1000ng/mL | 1nM | 6764±299.3 | 72.8±1 |
| 1000ng/mL | 10nM | 15445±347.1 | 83.9±0.2 |
| 1000ng/mL | 100nM | 18805±164.4 | 83.6±0.3 |
| 1000ng/mL | 1μM | 19018±379 | 83.8±0.4 |
| 1000ng/mL | 10μM | 19760±789.1 | 85.1±0.6 |

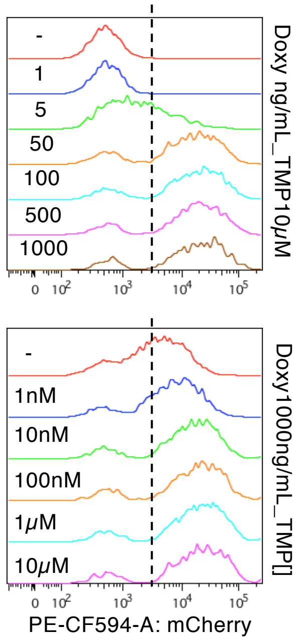


**Supplementary Figure 2**

**y**

| HeLa_EF1a-rtTA TRE3G-mCherry | Dynamic Time-Course | |
|---|---|---|
| **Doxy** | **Median Fluorescence Intensity (MFI)** | **% mCherry+ Cells** |
| - | 584.5±9.5 | 3.17±1.83 |
| 14hrs 100ng/mL | 3399.5±196.5 | 54.3±0.2 |
| 5hrs wash-out | 5107±127 | 72.25±2.15 |
| 8hrs wash-out | 4724±112 | 69.55±1.25 |
| 24hrs wash-out | 2565.5±96.5 | 44.25±2.55 |

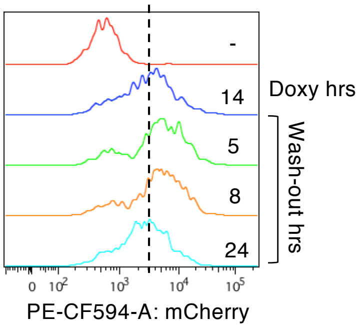


**z**

| HeLa_EF1a-rtTA TRE3G-DDmCherry | | Dynamic Time-Course | |
|---|---|---|---|
| **Doxy** | **TMP** | **Median Fluorescence Intensity (MFI)** | **% mCherry+ Cells** |
| - | 10nM | 405.7±13.1 | 0.77±0.16 |
| 14hrs 100ng/mL | 10nM | 5414±99 | 65.3±0.35 |
| 5hrs wash-out | | 4062±18.2 | 59.8±0.14 |
| 8hrs wash-out | | 2560±41.3 | 45.7±0.7 |
| 24hrs wash-out | | 769±3.9 | 5.1±0.07 |

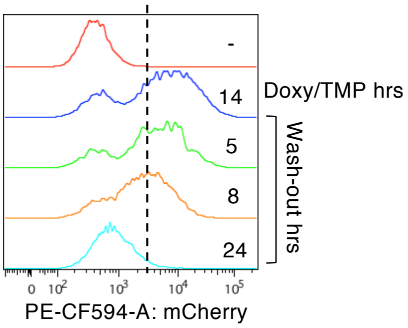


**Supplementary Figure 2**

**Supplementary Figure 2. Flow cytometry profiling, dose response, switch-off dynamics, single cell analysis and dynamic range of inducible cells.**

(a-d, p-s, x-z) Median Fluorescence Intensity (MFI) and % of mCherry+ cells measured by flow cytometry in EF1a-rtTA_TRE3G-mCherry (a, c), EF1a-rtTA_TRE3G-DDmCherry (b, d), EF1a-DDrtTA_TRE3G-mCherry (p, r), EF1a-DDrtTA_TRE3G-DDmCherry (q, s) mESCs, HeLa_EF1a-rtTA_TRE3G-mCherry (y) and HeLa_EF1a-rtTA_TRE3G-DDmCherry (x, z) cells. Inducer titration (a, b, p, q, x) and dynamic (c, d, r, s, y, z) response were performed using the concentrations and incubation times indicated in the tables. (e, f) Representative flow cytometry histograms of the dynamic switch-off analysed in 13 single clones sorted from EF1a-rtTA_TRE3G-mCherry (e) and EF1a-rtTA_TRE3G-DDmCherry (f) mESCs. (g) Box-plot representing mCherry+ cells in EF1a-rtTA_TRE3G-mCherry and EF1a-rtTA_TRE3G-DDmCherry single clones in a time-course experiment of 38hrs, in which inducers were washed out after incubation in the first 14hrs. The coefficient of variation, calculated on the mCherry+ cells, is also shown (g, inset). (h, i) Dual input regulation system consisting of the conditionally destabilised transactivator (DDrtTA) and a stable (h) or a conditionally destabilised (i) mCherry fluorescent protein. Protein expression following 24hrs Doxy/TMP treatment (1000ng/mL and 100nM, respectively) in EF1a-DDrtTA_TRE3G-mCherry (h, right panel) and EF1a-DDrtTA_TRE3G-DDmCherry (i, right panel) mESCs. (j-m) Fitted model simulations (dashed lines) and experimental data (dots) of EF1a-DDrtTA_TRE3G-mCherry (j, k, RMSE 0.0449 and 0.0669, respectively) and EF1a-DDrtTA_TRE3G-DDmCherry (l, m, RMSE 0.0907 and 0.084, respectively) mESC steady-state response. Dots represent experimental data of MFI in Supplementary Fig. 2p, q, normalised over the maximum activation point. (n, o) Model

predicted dynamic response (dashed lines) and experimental data (dots) of EF1a-DDrtTA_TRE3G-mCherry (n, RMSE 0.1195) and EF1a-DDrtTA_TRE3G-DDmCherry (o, RMSE 0.1916) mESCs. Dots represent experimental data of MFI in Supplementary Fig. 2r, s, normalised over the maximum activation point. (t-w) Simulations of the model (t, v) and experimental validation (u, w) of EF1a-DDrtTA_TRE3G-mCherry (t, u) and EF1a-DDrtTA_TRE3G-DDmCherry (v, w) mESC steady-state response following 24hrs induction with constant concentration of Doxy (100ng/mL) and varying TMP (0, 10nM; 10μM) or constant TMP (10nM) and varying Doxy (0, 50, 1000ng/mL). Maximum concentrations of Doxy and TMP (1000mg/mL and 10μM, respectively) are used as control. mCherry values are shown as heatmaps of scaled values across the entire dynamical range of expression levels. Data are means ±SEM (n=3, a-d, n-s, u, x, z; n=13, g; n=2, w, y); ±SD (n=3, j-m). Source data are provided as a Source Data file.

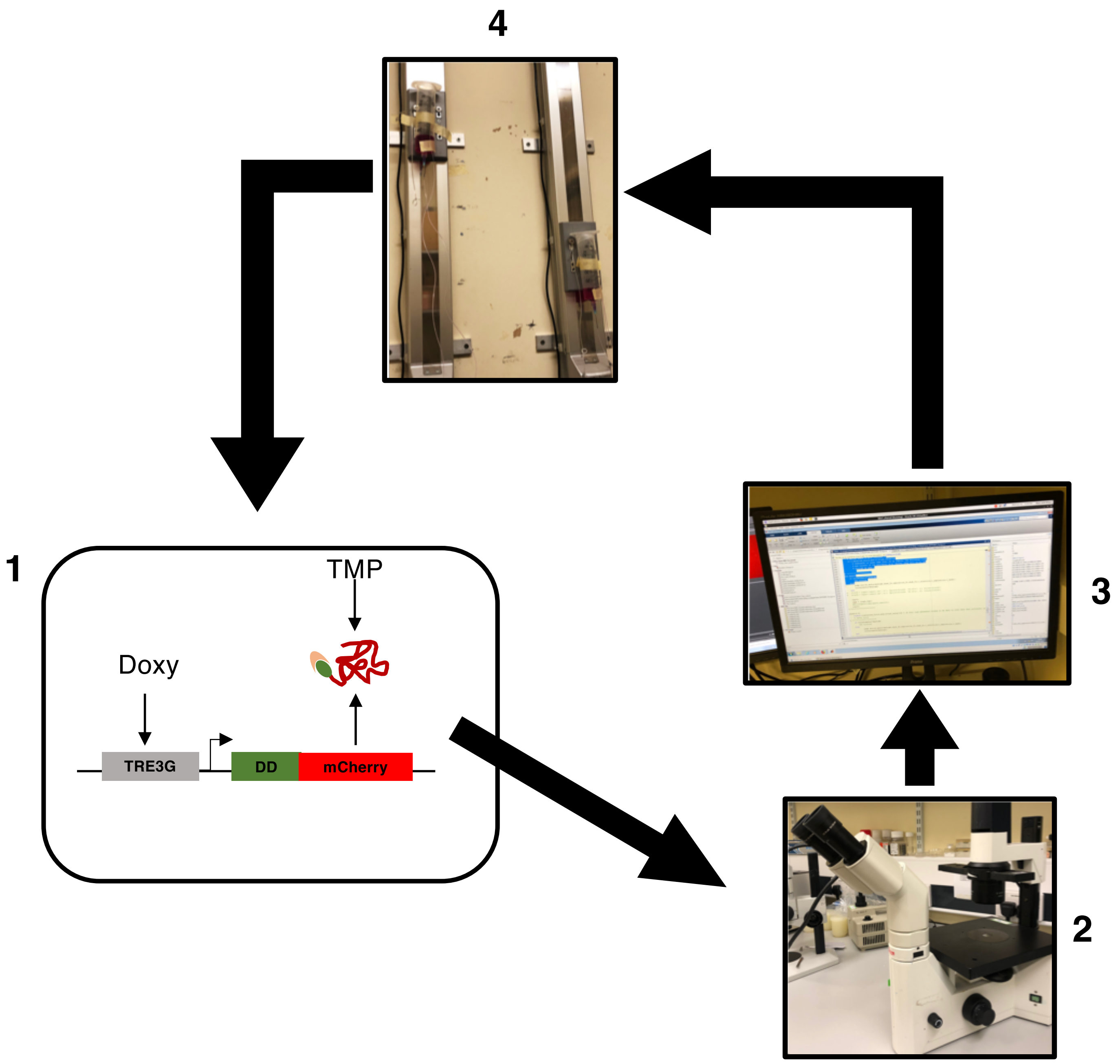


**Supplementary Figure 3**

**Supplementary Figure 3. *In-silico* feedback control microfluidics/microscopy platform**

Schematic representation of the *in-silico* feedback control platform used in the present study. It consists of the controlled biological system (1), in our case mouse Embryonic Stem Cells (mESCs) stably carrying an inducible exogenous gene (for specific control inputs used in different experiments see Supplementary Movies' legends and Main text); an inverted fluorescence microscope (2) measuring fluorescence every 60mins during the time-lapse; a computer (3) to implement real-time cell segmentation, fluorescence quantification and a Relay control algorithm (the latter computing the error from the comparison of the desired over the measured fluorescence); an actuation system (4), consisting of motor-controlled syringes dispensing media +/- inducers (+ inducers if the measured fluorescence is below the set-point and vice versa)[1].

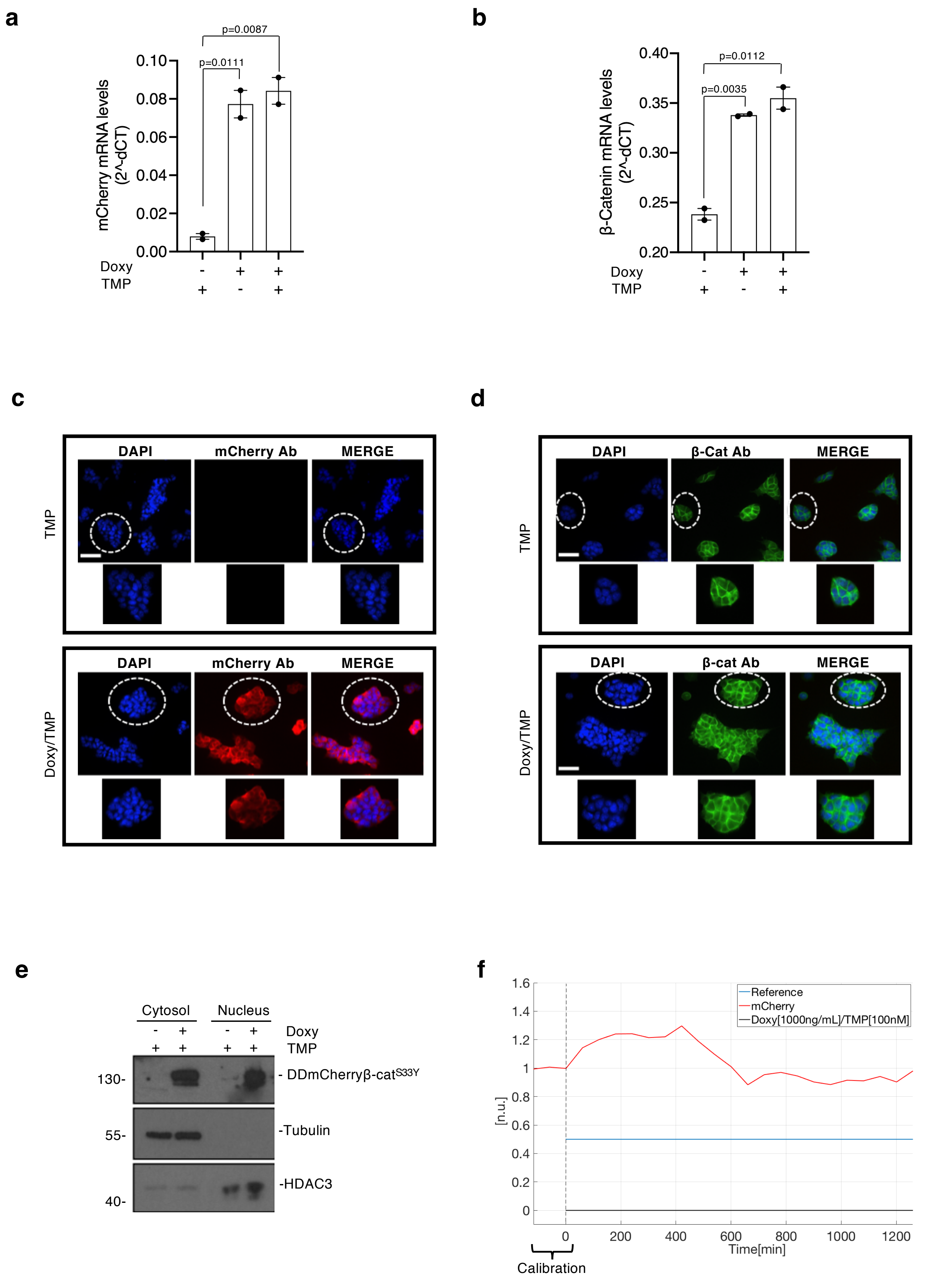


**Supplementary Figure 4**

g

| EF1a-rtTA TRE3G-DDmCherryβ-cateninS33Y | | Doxy/TMP Titration | |
|---|---|---|---|
| Doxy | TMP | Median Fluorescence Intensity (MFI) | % mCherry+ Cells |
| - | 10μM | 210.5±2.5 | 0.38±0.01 |
| 1ng/mL | 10μM | 217.5±0.5 | 0.34±0.06 |
| 5ng/mL | 10μM | 336.5±3.5 | 13.05±0.95 |
| 50ng/mL | 10μM | 1837.5±17.5 | 69.05±0.15 |
| 100ng/mL | 10μM | 2053.5±4.5 | 71.65±0.05 |
| 500ng/mL | 10μM | 1446±25 | 61.5±0.3 |
| 1000ng/mL | 10μM | 1257.5±7.5 | 57.35±0.25 |
| 1000ng/mL | - | 604.5±9.5 | 35.3±0.3 |
| 1000ng/mL | 1nM | 720.5±42.5 | 38.55±2.45 |
| 1000ng/mL | 10nM | 1146.5±52.5 | 53.75±1.65 |
| 1000ng/mL | 100nM | 1293.5±45.5 | 57.55±0.65 |
| 1000ng/mL | 1μM | 1374±14 | 59.8±0 |
| 1000ng/mL | 10μM | 1306±43 | 58.15±1.25 |

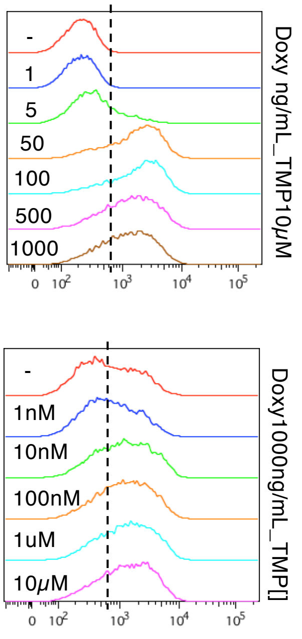


h

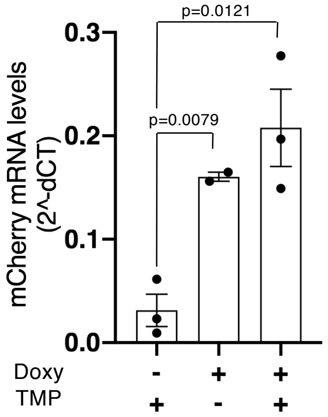


i

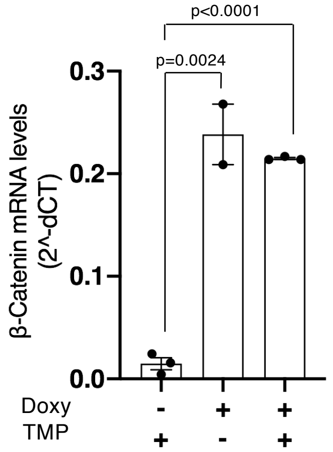


j

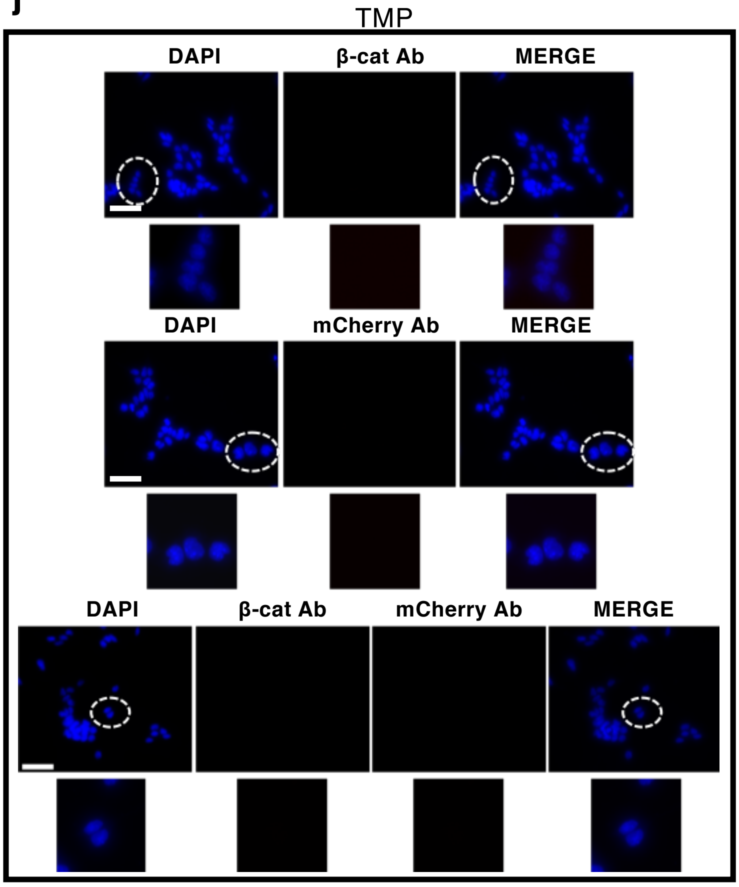


k

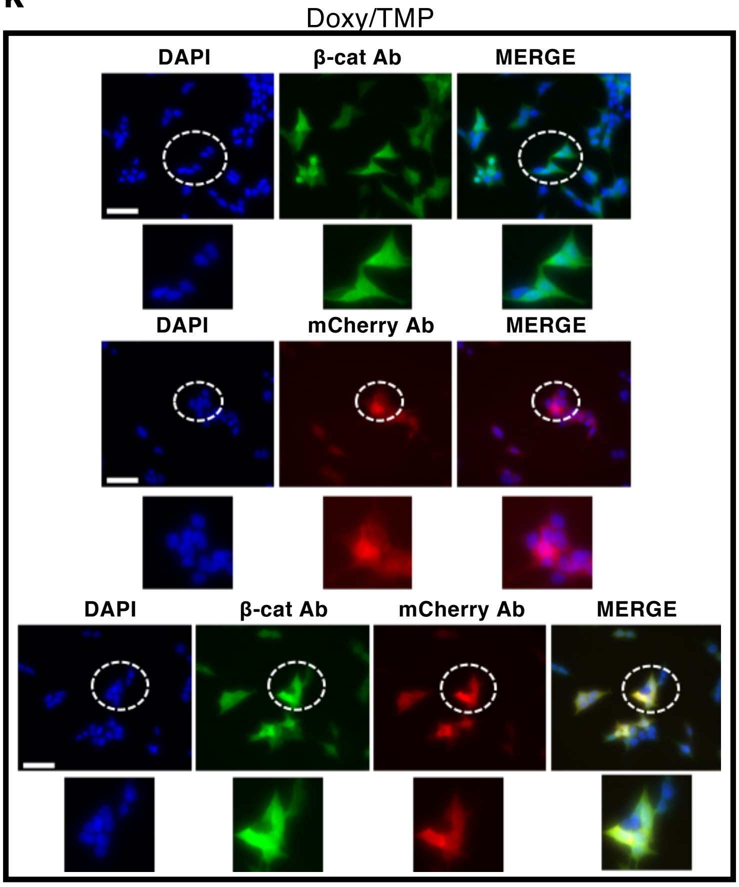


Supplementary Figure 4

**l**

| C1-EF1a-rtTA TRE3G-DDmCherryβ-cateninS33Y | | Doxy/TMP Titration | |
|---|---|---|---|
| **Doxy** | **TMP** | **Median Fluorescence Intensity (MFI)** | **% mCherry+ Cells** |
| - | 10$\mu$M | 246.5±6.5 | 0.6±0.05 |
| 1ng/mL | 10$\mu$M | 241±56 | 0.65±0.33 |
| 5ng/mL | 10$\mu$M | 476±4 | 21.3±0.1 |
| 50ng/mL | 10$\mu$M | 2683.5±82.5 | 81.7±1 |
| 100ng/mL | 10$\mu$M | 2754.5±55.5 | 80.1±1.2 |
| 500ng/mL | 10$\mu$M | 3105±221 | 84.3±1.6 |
| 1000ng/mL | 10$\mu$M | 2559.5±0.5 | 79.75±0.25 |
| 1000ng/mL | - | 2261±25 | 74.4±0.2 |
| 1000ng/mL | 1nM | 3094.5±477.5 | 81.1±2.3 |
| 1000ng/mL | 10nM | 3750.5±583.5 | 85.4±2.3 |
| 1000ng/mL | 100nM | 4115±129 | 87.5±0.2 |
| 1000ng/mL | 1$\mu$M | 4216±310 | 87.2±0.9 |
| 1000ng/mL | 10$\mu$M | 3836±391 | 86.55±1.15 |

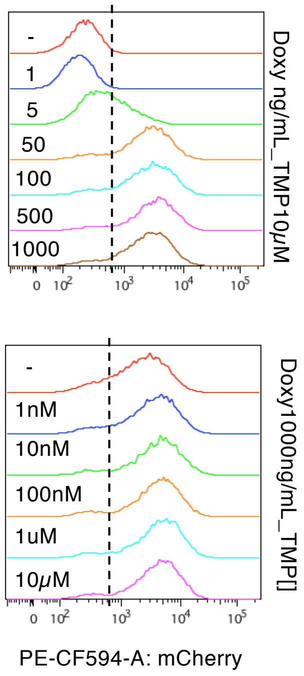


**m**

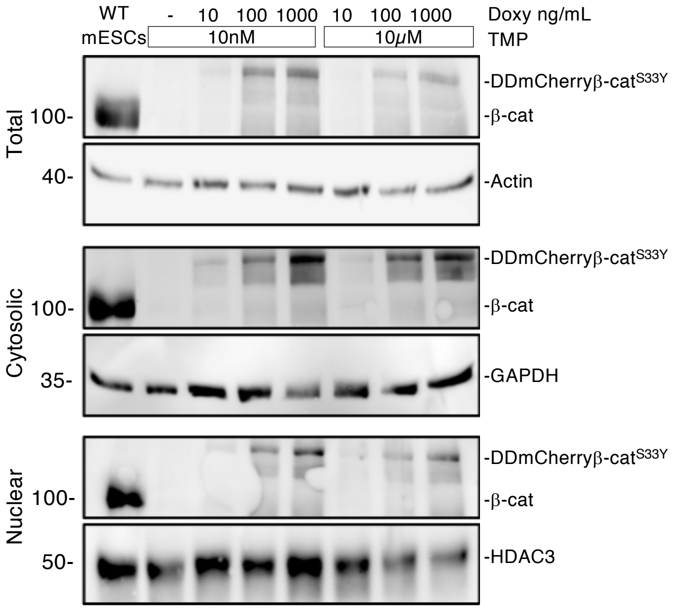


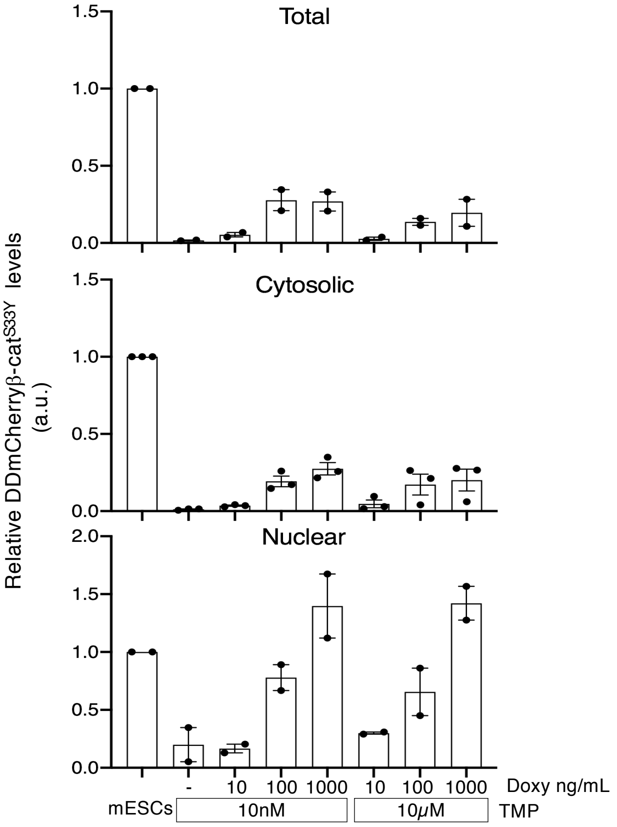


**n**

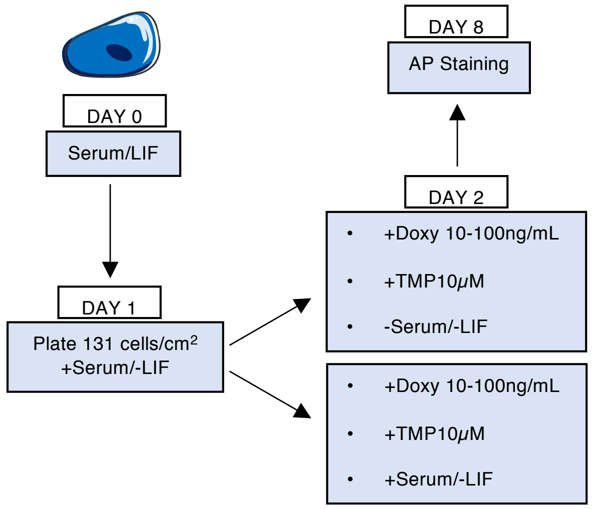


**o**

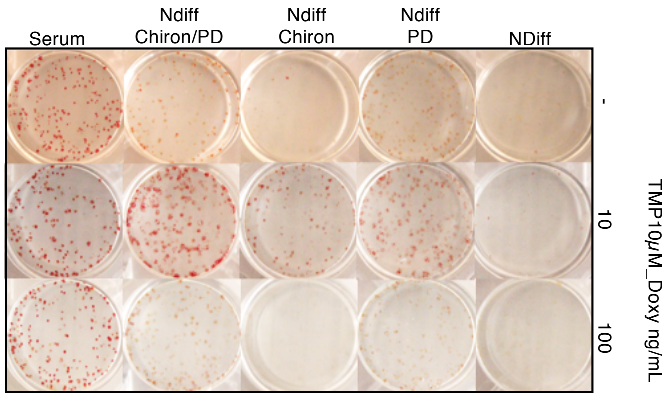


**Supplementary Figure 4**

p

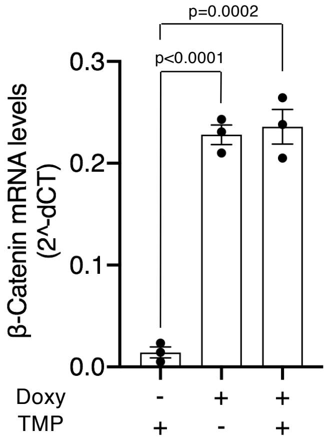


q

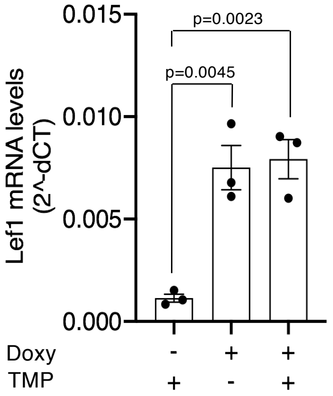


r

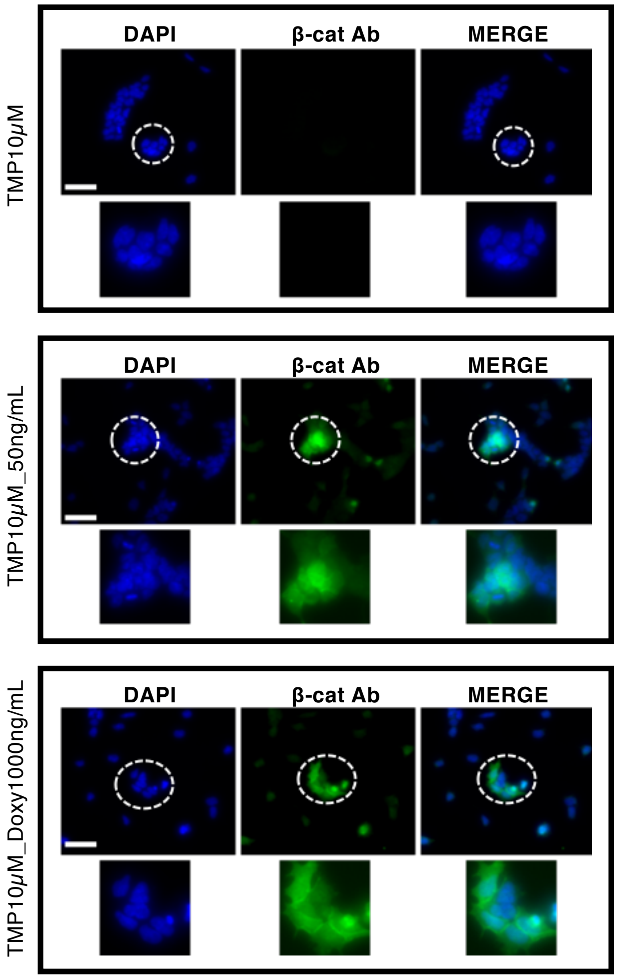


s

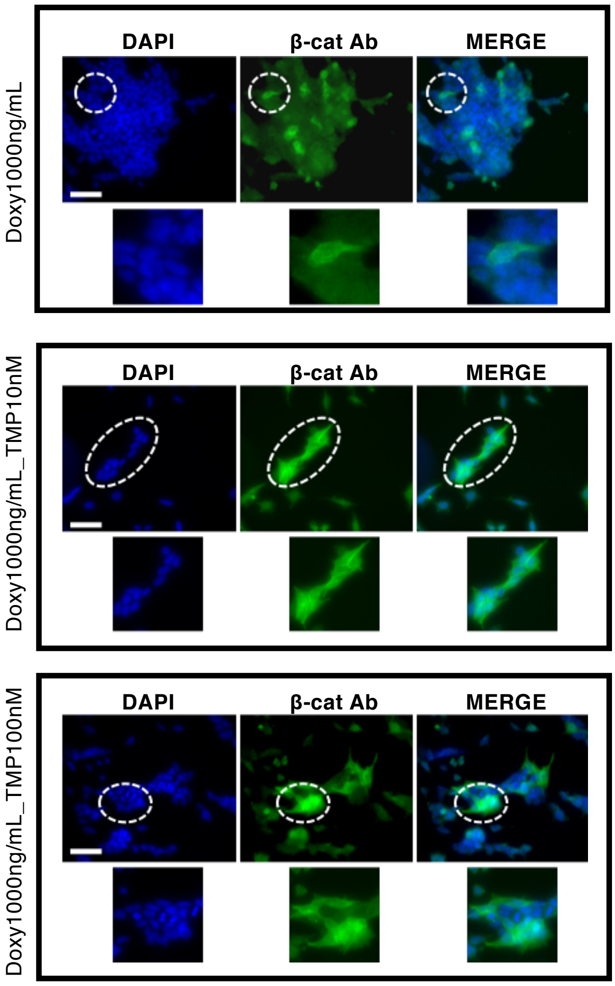


**Supplementary Figure 4**

t

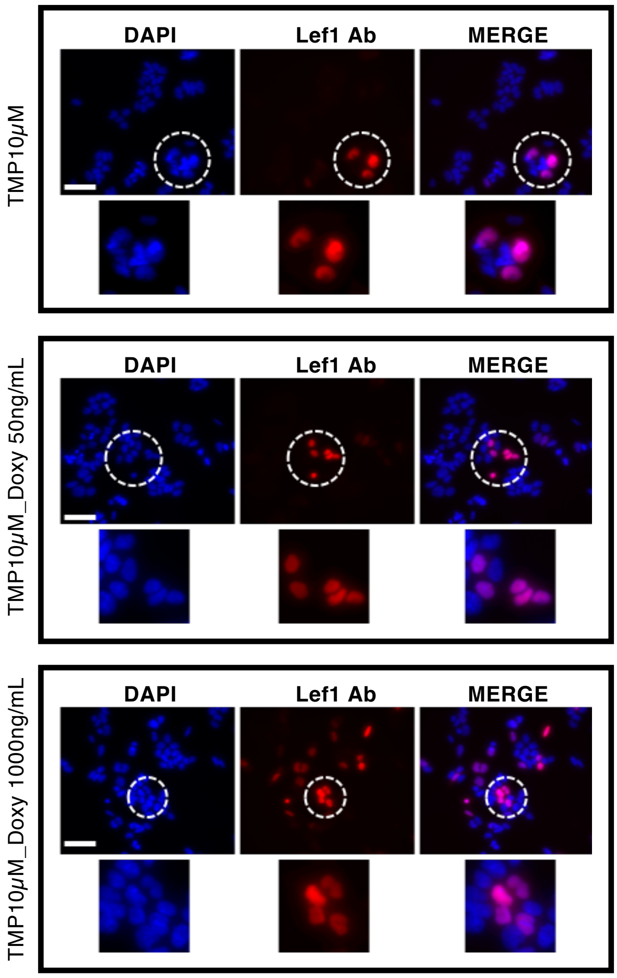


u

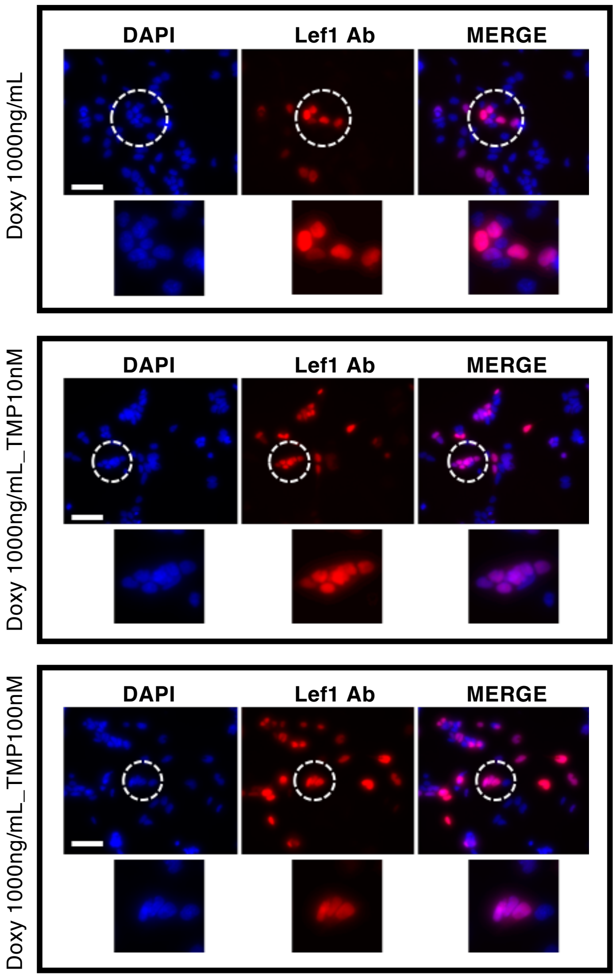


Supplementary Figure 4

**Supplementary Figure 4. Cell line characterisation and automatic control of Wnt/β-catenin pathway genes**

(a, b, h, i) DDmCherryβ-catenin$^{S33Y}$ mRNA levels measured by qPCR using mCherry (a, h) and β-catenin (b, i) specific primers in EF1a-rtTA_TRE3G-DDmCherryβ-catenin$^{S33Y}$ (a, b) and C1-EF1a-rtTA_TRE3G-DDmCherryβ-catenin$^{S33Y}$ (h, i) mESCs induced with Doxy (1000ng/mL) and/or TMP (100nM) for 24hrs. (c, d) β-catenin immunostaining in EF1a-rtTA_TRE3G-DDmCherryβ-catenin$^{S33Y}$ mESCs treated for 24hrs with Doxy (1000ng/mL) and/or TMP (100nM); mCherry (c) and β-catenin (d) antibodies were used. DAPI was used to stain the nuclei. Zoomed pictures of selected cells are shown. Scale bars 25μm. (e) Western-blot of nuclear and cytosolic fractions from EF1a-rtTA_TRE3G-DDmCherryβ-catenin$^{S33Y}$ mESCs treated for 24hrs with Doxy (1000ng/mL) and/or TMP (100nM), and blotted with an anti-mCherry antibody. (f) Set-point control experiment using inducers (Doxy 1000ng/mL and TMP 100nM) as control inputs in EF1a-rtTA_TRE3G-DDmCherryβ-catenin$^{S33Y}$ cells. In red the measured output (normalised mCherry fluorescence), in blue the control reference fluorescence, set at 50% of the average value measured during the calibration phase (120mins with continuous Doxy/TMP administration). The control performance indexes are reported in Supplementary Table 3 and Note 2; details about feedback control implementation are in Supplementary Note 2. (g, l) Median Fluorescence Intensity (MFI) and % of mCherry+ cells measured by flow cytometry in EF1a-rtTA_TRE3G-DDmCherryβ-catenin$^{S33Y}$ (g) and C1-EF1a-rtTA_TRE3G-DDmCherryβ-catenin$^{S33Y}$ (l) mESCs after 24hrs of treatment with indicated concentrations of Doxy and TMP. (j, k) β-catenin immunostaining in C1-EF1a-rtTA_TRE3G-DDmCherryβ-catenin$^{S33Y}$ mESCs treated for 24hrs with the indicated concentrations of TMP (j), or Doxy and TMP (k), using

mCherry (red signal) and β-catenin (green signal) antibodies. DAPI was used to stain the nuclei. Zoomed pictures of selected clones are shown. Scale bars 25μm. (m) Western-blot of total, cytosolic and nuclear fractions from C1-EF1a-rtTA_TRE3G-DDmCherryβ-catenin$^{S33Y}$ mESCs treated for 24hrs with TMP 10nM or 10μM and increasing concentration of Doxy (0, 10, 100, 1000ng/mL) and blotted with an anti-β-catenin antibody. Wild-type mESCs were used as control. Western-blot densitometric quantifications are shown (m, inset). Total, Cytosolic and Nuclear DDmCherryβ-catenin$^{S33Y}$ values are normalised against the corresponding housekeeping gene Actin, GAPD and HDAC3. (n) Clonogenicity assay experimental scheme. At Day 1, 131cells/cm$^2$ C1-EF1a-rtTA_TRE3G-DDmCherryβ-catenin$^{S33Y}$ mESCs were plated in complete mESCs growth media without LIF (+Serum/-LIF); the following day, inducers were added (TMP10μM and Doxy at 0, 10, 100ng/mL) and cells were either grown in Serum/-LIF or -Serum/-LIF supplemented with Chiron and/or PD media for 6 days; inducers were refreshed every 72hrs. At Day 8, cells were fixed and the Alkaline Phosphatase (AP) staining was performed. (o) Representative AP pictures from C1-EF1a-rtTA_TRE3G-DDmCherryβ-catenin$^{S33Y}$ mESCs treated with TMP10μM and Doxy (0, 10, 100ng/mL). The media used were either serum-based (Serum) or Serum-free (NDiff-Chiron/PD, NDiff-Chiron, NDiff-PD and NDiff). Red spots represent AP+ pluripotent colonies. (p, q) Exogenous DDβ-catenin$^{S33Y}$ (p) and DDLef1 (q) mRNA levels measured by qPCR in EF1a-rtTA_TRE3G-DDβ-catenin$^{S33Y}$ (p) and EF1a-rtTA_TRE3G-DDLef1 (q) mESCs. (r-u) β-catenin (r, s) and Lef1 (t, u) immunostaining in EF1a-rtTA_TRE3G-DDβ-catenin$^{S33Y}$ (r, s) and EF1a-rtTA_TRE3G-DDLef1 (t, u) mESCs treated for 24hrs with the indicated inducer concentrations. DAPI was used to stain the nuclei. Zoomed pictures of selected cells are shown. Scale bars 25μm.

Data are means ± SEM (n=2, a, b, g, l, m, inset; n=3, h, i, p, q). p values from two-tailed unpaired t test are shown. Source data are provided as a Source Data file.

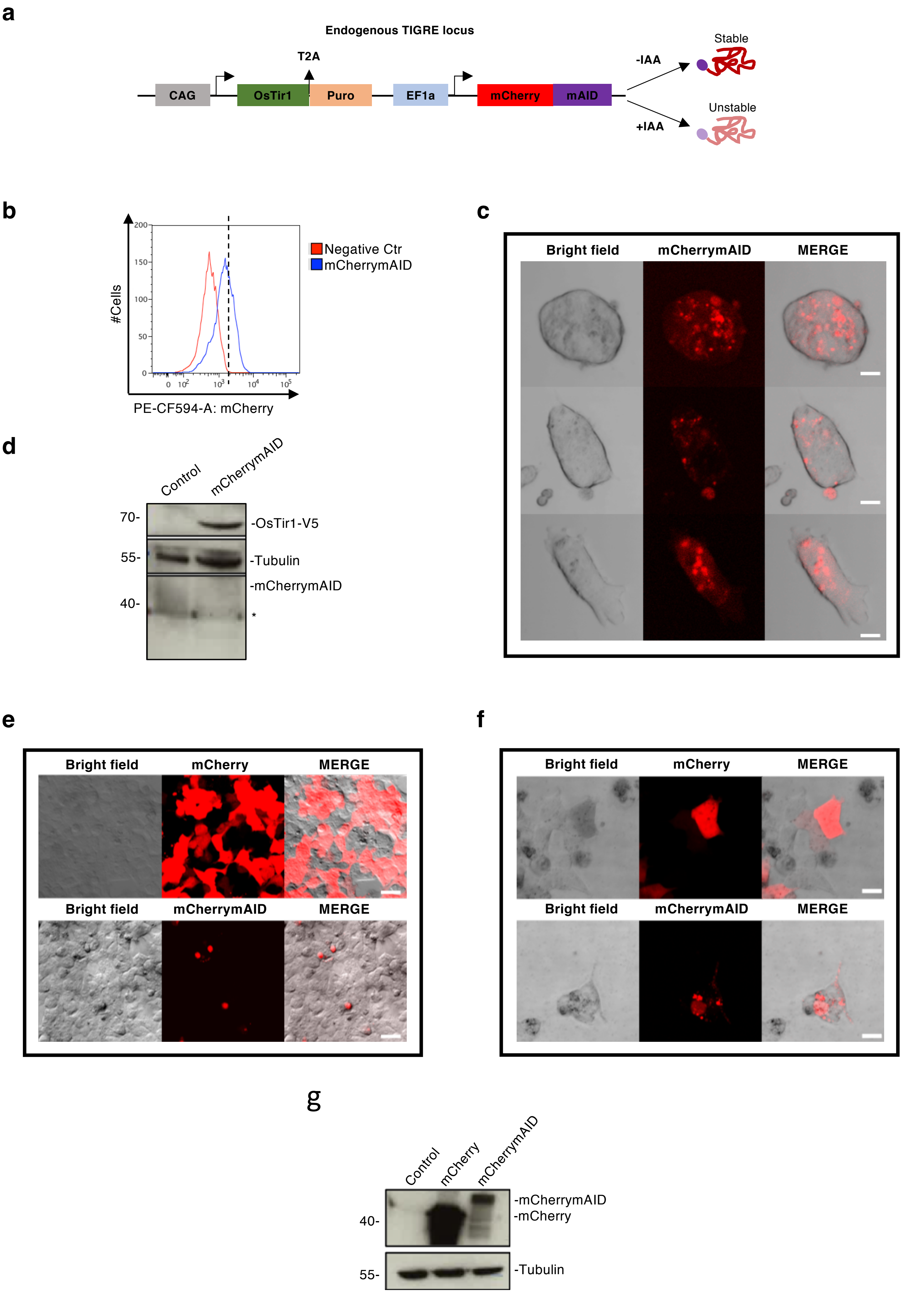


**Supplementary Figure 5**

**Supplementary Figure 5. AID (auxin-inducible degradation) system characterisation**

(a) The AID-system consisting of the auxin responsive F-box protein, Tir1 (CAG-OsTir1- V5-2A-PuroR) and the mini-AID fused mCherry (EF1a-mCherrymAID). (b) Representative flow cytometry profiles of negative control and mCherrymAID mESCs. (c) Confocal microscopy images of mCherrymAID mESCs. (d) mCherry and OsTIR1 protein levels measured in mCherrymAID mESCs by western-blot using anti-mCherry and anti-V5-tagged OsTIR1 antibodies. * indicates a non-specific band. (e, f) Confocal microscopy images of HEK293T cells transiently transfected with mCherry or mCherrymAID using equal (e) or cell-line adjusted (i.e. higher exposure in mCherrymAID cells, f) laser settings. (g) mCherry protein levels measured in mCherry and mCherrymAID HEK293T cells by western-blot using an anti-mCherry antibody. Scale bars 10μm. Source data are provided as a Source Data file.

**Supplementary Note 1: Mathematical modelling**

The mathematical models for the EF1a-rtTA_TRE3G-mCherry, EF1a-rtTA_TRE3G-DDmCherry, EF1a-DDrtTA_TRE3G-mCherry and EF1a-DDrtTA_TRE3G-DDmCherry systems are based on sets of 3 ODEs, describing transactivator protein, and fluorescent gene mRNA and protein concentrations, as the result of a production term and a degradation term. We formulated the parameters estimation problem as a constrained optimization problem:

$$\min_{\gamma} J(x, u; p)$$

$$s.t.:$$
$$\dot{x} = f(x, u; p)$$
$$p \geq 0$$

where $x$ is the state vector, $u$ is the vector of inputs acting on the systems (e.g. Doxy and TMP), $f$ is the vector field of the system dynamics and $p$ is the vector of all parameter to be identified. $J$ is the cost function to be minimized, defined as

$$J = \sum_{i=1}^{n} \left(\frac{y_i - \hat{y}_i}{\hat{y}_i}\right)^2$$

where $n$ is the number of experimental data points, $y$ are the predicted values of the mathematical model and $\hat{y}$ are the experimental data points used for fitting.

For the parameters identification, we used the Interior Point algorithm implemented in *fmicon* function (Matlab Optimization toolbox™, MathworksMatlab R2018a).

The parameters were first identified for EF1a-rtTA_TRE3G-mCherry and EF1a-rtTA_TRE3G-DDmCherry mESCs (equations (1)-(3), and (4)-(6), respectively). The Doxy and TMP Michaelis-Menten constants ($K_1$ and $K_3$, respectively) were directly fixed from dose-response experimental data (Fig. 2a-c), and the degradation of mCherry protein ($d_3$) was fitted to have mCherry protein half-life in range to that

measured experimentally (Fig. 1f; Supplementary Fig.1a). The remaining parameters were fitted as aforementioned, with a constrain on the term describing the degradation of DDmCherry protein in equation (6) $\left(d_3 + d_4\left(\frac{K_3^{h_3}}{K_3^{h_3}+TMP^{h_3}}\right)\right)$ so that, in presence of saturating TMP concentration, the DDmCherry protein half-life would be equal to that of untagged mCherry protein.

For the EF1a-DDrtTA_TRE3G-mCherry and EF1a-DDrtTA_TRE3G-DDmCherry systems (equations (7)-(9), and (10)-(12), respectively), the Hill coefficients for Doxy ($h_1$) and for TRE3G promoter ($h_2$), the TRE3G Michaelis-Menten constant ($K_2$) and degradation rate of mCherry mRNA ($d_3$) were fixed equal to the corresponding parameter values identified for the EF1a-rtTA systems. The Doxy and TMP Michaelis-Menten constants ($K_1$ and $K_3$, respectively) were fixed from dose-response experimental data (Supplementary Fig. 2j-m). For EF1a-DDrtTA_TRE3G-DDmCherry mESCs, parameters related to TMP-dependent degradation were kept identical in the transactivator and DDmCherry protein equations. The remaining parameters were fitted as aforementioned, with a constrain on TMP-dependent degradation of the transactivator and DDmCherry proteins $\left(d_1 + d_2\left(\frac{K_3^{h_3}}{K_3^{h_3}+TMP^{h_3}}\right)\right)$ and $\left(d_4 + d_2\left(\frac{K_3^{h_3}}{K_3^{h_3}+TMP^{h_3}}\right)\right)$, respectively, so that, in presence of saturating TMP concentration, the degradation rates would be equal to those of the corresponding untagged proteins.

Letting $x_1$ be the rtTA (DD-tagged or not) protein concentration, $x_2$ the mCherry mRNA concentration and $x_3$ the mCherry (DD-tagged or not) protein concentration, the models of the systems considered in this work are reported below. The description of the estimated parameters and their values can be found in Supplementary Tables 1

and 2. Of note, variables concentrations are in [a.u.], as the model was fitted on normalised FACS data; the latter was used for model fitting or validation as described in the main text. The Root Mean Squared error (RMSE) reported to quantify goodness of fitting and predictions was calculated using the formula $\sqrt{\frac{\sum_{i=1}^{n}(y_i-\hat{y}_i)^2}{n}}$ where $n$ is the number of experimental data points, $y$ are the predicted values of the mathematical model and $\hat{y}$ are the experimental data points used for fitting.

**EF1a-rtTA_TRE3G-mCherry model**

$$\frac{dx_1}{dt} = \gamma_1 - d_1x_1 \text{ (1)}$$

$$\frac{dx_2}{dt} = \alpha_1 + \alpha_2\left(\frac{x_1^{h_2}\left(\frac{Dox^{h_1}}{K_1^{h_1}+Dox^{h_1}}\right)}{K_2^{h_2} + x_1^{h_2}\left(\frac{Dox^{h_1}}{K_1^{h_1}+Dox^{h_1}}\right)}\right) - d_2x_2 \text{ (2)}$$

$$\frac{dx_3}{dt} = \gamma_2x_2 - d_3x_3 \text{ (3)}$$

**EF1a-rtTA_TRE3G-DDmCherry model**

$$\frac{dx_1}{dt} = \gamma_1 - d_1x_1 \text{ (4)}$$

$$\frac{dx_2}{dt} = \alpha_1 + \alpha_2\left(\frac{x_1^{h_2}\left(\frac{Dox^{h_1}}{K_1^{h_1}+Dox^{h_1}}\right)}{K_2^{h_2} + x_1^{h_2}\left(\frac{Dox^{h_1}}{K_1^{h_1}+Dox^{h_1}}\right)}\right) - d_2x_2 \text{ (5)}$$

$$\frac{dx_3}{dt} = \gamma_2x_2 - d_3x_3 - d_4\left(\frac{K_3^{h_3}}{K_3^{h_3} + TMP^{h_3}}\right)x_3 \text{ (6)}$$

**Supplementary Table 1:** Parameters identified for the EF1a-rtTA_TRE3G-mCherry and EF1a-rtTA_TRE3G-DDmCherry systems.

| Parameter | Description | Value |
|---|---|---|

| | | |
|---|---|---|
| $\gamma_1$[a.u. hrs$^{-1}$] | Production rtTA protein | 54.61 |
| $d_1$[hrs$^{-1}$] | Degradation rate rtTA protein | 55.12 |
| $\alpha_1$ [a.u.hrs$^{-1}$] | Basal activity TRE3G | 0.58 |
| $\alpha_2$ [a.u.hrs$^{-1}$] | Maximal transcription rate TRE3G | 22.80 |
| $K_1$ [ng/mL] EF1a-tTA_TRE3G-mCherry | Doxy Michaelis-Menten constant | 64.00 |
| $K_1$ [ng/mL] EF1a-tTA_TRE3G-DDmCherry | Doxy Michaelis-Menten constant | 87.07 |
| $K_2$ [a.u.] | TRE3G Michaelis-Menten constant | 0.91 |
| $h_1$ | Hill coefficient for Doxy | 2.34 |
| $h_2$ | Hill coefficient of the TRE3G promoter | 6.21 |
| $d_2$[hrs$^{-1}$] | Degradation rate mCherry mRNA | 72.09 |
| $\gamma_2$[hrs$^{-1}$] | Production rate mCherry protein | 0.57 |
| $d_3$[hrs$^{-1}$] | Degradation rate mCherry protein | 0.11 |
| $d_4$[hrs$^{-1}$] | TMP-dependent degradation rate DDmCherry protein | 0.40 |
| $K_3$ [ng/mL] | TMP Michaelis-Menten constant | 0.40 |
| $h_3$ | Hill coefficient for TMP | 1.20 |

**EF1a-DDrtTA_TRE3G-mCherry model**

$$\frac{dx_1}{dt} = \gamma_1 - d_1x_1 - d_2\left(\frac{K_3^{h_3}}{K_3^{h_3} + TMP^{h_3}}\right)x_1 \quad (7)$$

$$\frac{dx_2}{dt} = \alpha_1 + \alpha_2\left(\frac{x_1^{h_2}\left(\frac{Dox^{h_1}}{K_1^{h_1}+Dox^{h_1}}\right)}{K_2^{h_2} + x_1^{h_2}\left(\frac{Dox^{h_1}}{K_1^{h_1}+Dox^{h_1}}\right)}\right) - d_3x_2 \quad (8)$$

$$\frac{dx_3}{dt} = \gamma_2x_2 - d_4x_3 \quad (9)$$

**EF1a-DDrtTA_TRE3G-DDmCherry model**

$$\frac{dx_1}{dt} = \gamma_1 - d_1 x_1 - d_2 \left( \frac{K_3^{h_3}}{K_3^{h_3} + TMP^{h_3}} \right) x_1 \quad (10)$$

$$\frac{dx_2}{dt} = \alpha_1 + \alpha_2 \left( \frac{x_1^{h_2} \left( \frac{Dox^{h_1}}{K_1^{h_1} + Dox^{h_1}} \right)}{K_2^{h_2} + x_1^{h_2} \left( \frac{Dox^{h_1}}{K_1^{h_1} + Dox^{h_1}} \right)} \right) - d_3 x_2 \quad (11)$$

$$\frac{dx_3}{dt} = \gamma_2 x_2 - d_4 x_3 - d_2 \left( \frac{K_3^{h_3}}{K_3^{h_3} + TMP^{h_3}} \right) x_3 \quad (12)$$

**Supplementary Table 2:** Parameters identified for the EF1a-DD::rtTA_TRE3G-mCherry and EF1a-DD::rtTA_TRE3G-DD::mCherry systems.

| Parameter | Description | Value |
|---|---|---|
| $\gamma_1$[a.u. hrs$^{-1}$] | Production rtTA protein | 0.37 |
| $d_1$[hrs$^{-1}$] | Degradation rate rtTA protein | 0.51 |
| $\alpha_1$[a.u.hrs$^{-1}$] | Basal activity TRE3G | 0.47 |
| $\alpha_2$ [a.u.hrs$^{-1}$] | Maximal transcription rate TRE3G promoter | 73.70 |
| $K_1$ [ng/mL] EF1a-DDrtTA tTA_TRE3G-mCherry/ TRE3G-DDmCherry | Doxy Michaelis-Menten constant | 103 |
| $K_2$ [a.u.] | TRE3G Michaelis-Menten constant | 0.91 |
| $h_1$ | Hill coefficient for Doxy | 2.34 |
| $h_2$ | Hill coefficient of the TRE3G promoter | 6.21 |
| $d_3$[hrs$^{-1}$] | Degradation rate mCherry mRNA | 72.09 |
| $\gamma_2$[hrs$^{-1}$] | Production rate mCherry protein | 0.83 |
| $d_4$[hrs$^{-1}$] | Degradation rate mCherry protein | 0.161 |
| $d_2$[hrs$^{-1}$] | TMP-dependent degradation rate | 0.14 |

| | | |
|---|---|---|
| | DDmCherry protein and DDrtTA protein | |
| $K_3$ [ng/mL] | TMP Michaelis-Menten constant | 0.70 |
| $h_3$ | Hill coefficient for TMP | 1.05 |

**Supplementary Note 2: Microfluidics/microscopy-based time lapses**

Microfluidics/microscopy-based experiments were performed using the device designed and optimised for mammalian cells in the laboratory of Prof Jeff Hasty at the University California in San Diego[1,2]. The topology of the device ensures controlled flow perfusion, minimised cell stress and controlled $CO_2$ diffusion. As described in[1], cells from a sub-confluent 60cm petri dished were washed with sterile Phosphate-Buffered Saline (PBS, Gibco), trypsinised for 2-3' at room temperature and centrifuged at 8000 xg for 5'. Pelleted cells were resuspended in 200uL of complete mESC media supplemented with Doxy/TMP (Figs. 3a-c, e, 4e and Supplementary Fig. 4f; Supplementary Movies 1-3, 5, 7, 8) or Doxy (Fig. 3d, f; Supplementary Movies 4, 6) and chip-loaded. Before loading, the chip was fulfilled with media containing Doxy/TMP (Figs. 3a-c, e, 4e and Supplementary Fig. 4f; Supplementary Movies 1-3, 5, 7, 8) or Doxy (Fig. 3d, f; Supplementary Movies 4, 6) from port 5 first and port 1 after. Cell suspension was loaded from port 1 while the vacuum applied to ports 3 and 4. The vacuum allows air to be released from chambers, facilitating cell trapping. Cells were cultured for 24hrs in a tissue culture incubator (5%CO2, 37◦C) under constant perfusion with Doxy (Fig. 3d, f; Supplementary Movies 4, 6) or Doxy/TMP (Figs. 3a-c, e, 4e and Supplementary Fig. 4f; Supplementary Movies 1-3, 5, 7, 8) containing media. Media was perfused with a syringe directly connected to port 2 *via* 24-gauge PTFE tubing (Cole-Parmer Inc.). Port 5 was used for waste media whereas ports 1, 6 and 7

were plugged to avoid media spillage. The day after, the device was transferred on the widefield microscope for *in-silico* feedback control experiments. The actuation system consists of two motor-controlled syringes (http://biodynamics.ucsd.edu/dialawave/) connected to port 6 and 7. One syringe contains Doxy- (Fig. 3a, d, f; movies 1, 4, 6) or TMP- (Fig. 3b; Supplementary Movie 2) or Doxy/TMP- (Figs. 3c, e, 4e and Supplementary Fig. 4f; Supplementary Movies 3, 5, 7, 8) enriched media, whereas the other contains plain media (Figs. 3c-f, 4e and Supplementary Fig. 4f.; Supplementary Movies 3-8), or TMP- (Fig. 3a; Supplementary Movie 1) or Doxy- (Fig. 3b; Supplementary Movie 2) supplemented media, depending on experimental set-up. Ports 1, 2 and 5 are also connected to static syringes working as waste tanks. If the height of the waste tanks is fixed, the one of the perfusing media ports is automatically adjusted during the experiment to change the input provided to cells. Input administration was measured using a green dye (1μM Atto488 dye from ThermoFisher), added to Doxy/TMP or Doxy containing syringe.

*Image segmentation*

The segmentation algorithm follows the methodology illustrated in[1]. Briefly, a threshold is defined to generate a binary image selecting only pixels belonging to cell edges. Then, by using dilation and filling operators, it derives a binary image (mask) that selects the portion of the original image covered by cells. The mask obtained is applied to the red field image. In order to calculate the average intensity fluorescence of pixels belonging to cells, the background signal measured in a cell-free portion of the chamber is subtracted from the value of mask pixels.

*Fluorescence microscopy*

The control platform consists of a Leica DMi8 inverted microscope equipped with the digital camera AndoriXON 897 ultra back-illuminated EMCCD (512x512 16µm pixels, 16 bit, 56 fps at full frame), and an environmental control chamber (PeCon) for long-term temperature control and CO2 enrichment. The Adaptive Focus Control (AFC) ensures focus is maintained during the entire time-course experiment. The experimental set-up includes consecutive acquisition in three channels (phase contrast, green and red fluorescence) with a 20X objective every 60mins. Of note, for the control experiments in Fig. 3d, f; Supplementary Movies 4, 6, the power lamp was increased from 33 to 50% given the lower cell fluorescence of EF1a-rtTA_TRE3G-DDmCherry mESCs activated with Doxy only (Supplementary Fig. 2d, main text).

*Relay control*

The Relay control strategy can be expressed as follows:

$$u(t) = \begin{cases} u_{MAX} = 1 & if \quad e(t) > 0 \\ u_{MIN} = 0 & if \quad e(t) < 0 \end{cases}$$

where the control error $e(t) = r(t) - y(t)$ is the difference between the control reference signal $r$ and the system output $y$; $u$ is the control input. The Relay controller requires only the computation of the control error at each sampling time $e(kT)$, where $T = 60$ mins, whose sign dictates which input must be provided to the cells. Specifically, cells are treated with inducers-containing medium for the next 60mins if $e(kT) > 0$ or medium without inducer otherwise. A 5% hysteresis interval to the controller, corresponding to a tolerance interval around the set-point in which the Relay algorithm ignores the control error, was added to avoid chattering[1].

In control experiments, we controlled EF1a-rtTA_TRE3G-DDmCherry mESCs using differently control inputs: Doxy (1000ng/mL) in Fig. 3a, d, f; TMP (100nM) in Fig. 3b;

combination of Doxy (1000ng/mL) and TMP (100nM) in Fig. 3c, e and Supplementary Fig. 4f; combination of Doxy (100ng/mL) and TMP (10nM) in Fig. 4e.

The Relay control strategy, although being simple, succeeds in keeping the system output close to the desired static or varying reference. Typically, the controlled variable oscillates around the reference and it is acceptable if the oscillation amplitude is sufficiently small[3].

*Control Performance Indexes*

To measure the performance of the control strategies adopted, we considered the following metrics defined as integral expressions related to the control defined above.

1. The Integral of Squared Error (ISE)

$$ISE = \int_0^\infty [e(t)]^2 \, dt$$

ISE integrates the square of the error over time penalising large errors more than smaller ones.

2. The Integral Absolute Error (IAE)

$$IAE = \int_0^\infty |e(t)| \, dt$$

IAE integrates the absolute error over time without adding weight to any of the errors in a systems response.

3. The Integral Time-weighted Absolute Error (ITAE)

$$ITAE = \int_0^\infty t |e(t)| \, dt$$

ITAE integrates the absolute error multiplied by the time over time. It is a weighted version of IAE. It weights errors that exist after a long time much more heavily than those at the start of the response.

**Supplementary Table 3:** Parameter Indexes calculated for the control experiments in Figs. 3a-f (Supplementary Movies 1-6), 4e (Supplementary Movie 8) and Supplementary Fig. 4f (Supplementary Movie 7).

| **Figure/ Supplementary Movie** | **ISE** | **IAE** | **ITAE** |
|---|---|---|---|
| Fig. 3a, movie 1 | 6.56 | 10.2939 | 83.4885 |
| Fig. 3b, movie 2 | 3.2176 | 58.8588 | 7.5369 |
| Fig. 3c, movie 3 | 0.7159 | 2.3828 | 12.7121 |
| Fig. 3d, movie 4 | 0.3188 | 1.9998 | 14.3546 |
| Fig. 3e, movie 5 | 0.3772 | 1.9926 | 18.4638 |
| Fig. 3f, movie 6 | 1.0318 | 3.9879 | 42.6792 |
| Fig. 4e, movie 8 | 0.839 | 2.73 | 12.5834 |
| Supplementary Fig. 4f, movie 7 | 6.7844 | 11.5592 | 101.7628 |

**Supplementary references**

1 Fracassi, C., Postiglione, L., Fiore, G. & di Bernardo, D. Automatic Control of Gene Expression in Mammalian Cells. *ACS Synth Biol* **5**, 296-302, doi:10.1021/acssynbio.5b00141 (2016).

2 Kolnik, M., Tsimring, L. S. & Hasty, J. Vacuum-assisted cell loading enables shear-free mammalian microfluidic culture. *Lab Chip* **12**, 4732-4737, doi:10.1039/c2lc40569e (2012).

3 Murray, K. J. Å. R. M. *Feedback Systems: An Introduction for Scientists and Engineers*. (2010).
