# Supplementary material for: A tunable dual-input system for on-demand dynamic gene expression regulation
Source: Nat Commun. 2019 Oct 2;10:4481. doi: 10.1038/s41467-019-12329-9 (PMC6775159; doi:10.1038/s41467-019-12329-9)
Supplement: Supplementary file 10 — Description of Additional Supplementary Files [file 41467_2019_12329_MOESM10_ESM.doc]

**Description of Additional Supplementary Files**

**Supplementary movies. Microfluidics/microscopy-based in-silico feedback control of gene expression in mESCs**

Automatic control starts after a calibration phase of 120mins, during which cells are perfused with Doxy+/-TMP supplemented media to set maximal initial fluorescence. Cells are imaged every 60mins, and the segmented fluorescence (red line) is compared to the control reference (blue line). Finally, motor-controlled syringes are automatically switched, following execution of the control algorithm. Cells receive media with specific inputs when the fluorescence is above the reference and vice versa. Input concentration in the microfluidics device (black line, lower graph) is measured over time using a fluorescent dye.

File Name: Supplementary Movie 1

Description: Set-point DDmCherry transcriptional control. EF1artTA_TRE3G-DDmCherry mESCs are kept with constant perfusion of TMP (100nM)- enriched media, using Doxy (1000ng/mL) as control input. Cells are perfused with Doxy 1000ng/mL and TMP100nM in the tissue culture incubator over-night before the control experiment starts

File Name: Supplementary Movie 2

Description: Set-point DDmCherry post-translational control. EF1artTA_TRE3G-DDmCherry mESCs are kept with constant perfusion of Doxy (1000ng/mL)-enriched media, using TMP (100nM) as control input. Cells are perfused with Doxy 1000ng/mL and TMP100nM in the tissue culture incubator overnight before the control experiment starts

File Name: Supplementary Movie 3

Description: Set-point DDmCherry dual-input control. EF1artTA_TRE3G-DDmCherry mESCs are perfused either with plain media, or Doxy/TMP (control inputs, 1000ng/mL and 100nM, respectively)-enriched media. Cells are perfused with Doxy 1000ng/mL and TMP100nM in the tissue culture incubator overnight before the control experiment starts.

File Name: Supplementary Movie 4

Description: Set-point DDmCherry transcriptional control (no TMP induction). EF1a-rtTA_TRE3G-DDmCherry mESCs are perfused either with plain media, or Doxy (control inputs, 1000ng/mL)-enriched media. Cells are perfused with Doxy 1000ng/mL in the tissue culture incubator over-night before the control experiment starts

File Name: Supplementary Movie 5

Description: Multi set-point DDmCherry dual-input control. EF1artTA_TRE3G-DDmCherry mESCs are perfused either with plain media, or Doxy/TMP (control inputs, 1000ng/mL and 100nM, respectively)-enriched media. Cells are perfused with Doxy 1000ng/mL and TMP100nM in the tissue culture incubator overnight before the control experiment starts

File Name: Supplementary Movie 6

Description: Multi set-point DDmCherry transcriptional control. EF1a-rtTA_TRE3G-DDmCherry mESCs are perfused either with plain media, or Doxy (control inputs, 1000ng/mL)-enriched media. Cells are perfused with Doxy 1000ng/mL in the tissue culture incubator over-night before the control experiment starts.

File Name: Supplementary Movie 7

Description: Set-point DDmCherryb-cateninS33Y dual-input control. EF1a-rtTA_TRE3G-DDmCherryb-cateninS33YmESCs are perfused either with plain media, or Doxy/TMP (control inputs, 1000ng/mL and 100nM, respectively)-enriched media. Cells are perfused with Doxy 1000ng/mL and TMP100nM in the tissue culture incubator over-night before the control experiment starts.

File Name: Supplementary Movie 8

Description: Set-point DDmCherryb-cateninS33Y dual-input control. EF1a-rtTA_TRE3G-DDmCherryb-cateninS33YmESCs are perfused either with plain media, or Doxy/TMP (control inputs, 100ng/mL and 10nM, respectively)-enriched media. Cells are perfused with Doxy 100ng/mL and TMP10nM in the tissue culture incubator over-night before the control experiment starts.
